# Modified Couple Stress Theory for nonlinear primary resonance of FG-GPLRC cylindrical shells in thermal environment

**DOI:** 10.1371/journal.pone.0323442

**Published:** 2025-06-04

**Authors:** Mohammad Amin Forghani, Ramin Abdellahi, Mohsen Esmaeili, Roohallah Alizadehsani, Paweł Pławiak

**Affiliations:** 1 Department of Mechanical Engineering, Shiraz Branch Islamic Azad University, Shiraz, Iran; 2 Department of Mechanical Engineering, Khomeinishahr Branch, Islamic Azad University, Khomeinishahr, Iran; 3 Department of Mechanical Engineering, Islamic Azad University, Tehran, Iran; 4 Institute for Intelligent Systems Research and Innovation (IISRI), Deakin University, Australia; 5 Department of Computer Science, Faculty of Computer Science and Telecommunica-tions, Cracow Uni-versity of Technology, Krakow, Poland; 6 Institute of Theoretical and Applied Informatics, Polish Academy of Sciences, Gli-wice, Poland; Khalifa University of Science and Technology, UNITED ARAB EMIRATES

## Abstract

A modified couple stress theory (MCST)-based microshell model for functionally graded graphene platelets reinforced composite (FG-GPLRC) is proposed for the first time to investigate the nonlinear forced vibration behavior of reinforced microshells subjected to extreme temperatures. To achieve this, the effective elastic modulus is derived using the modified Halpin–Tsai model, while the rule of mixtures is applied for density, Poisson’s ratio, and thermal expansion coefficients. The first-order shear deformation theory (FSDT) and von Karman strains are considered, and nonlinear governing partial differential equations (PDEs) are derived using Hamilton’s principle, which accounts for size effects and initial stresses induced by the thermal environment. The Galerkin method, coupled with the multiple timescale method (MSM), is employed to solve the PDEs and obtain the nonlinear frequency-amplitude curve for primary resonance. The accuracy of the method is validated by comparison with previous research. The study examines the influence of GPL weight fraction, thickness distribution, temperature variations, geometric ratios, and material length scale parameters on the amplitude-frequency curves of nanocomposite cylindrical microshells. The results show that increasing the GPL content and the material length scale parameter leads to higher resonance frequencies. Additionally, while the small-scale parameter amplifies nonlinearity, an increase in the GPL content, especially near the inner and outer surfaces of the shell, reduces the nonlinearity of the reinforced composite. These findings provide valuable benchmarks for evaluating the performance of alternative methods.

## 1 Introduction

Due to the rapid advancements in the science and engineering of micro-scale structures subjected to dynamic loads and vibrations, it has become essential to investigate the dynamic behavior of micro-structures, such as cylindrical micro-gyroscopes [1] and microshell resonators [2], with consideration of the small-scale effects on their characteristics. Therefore, studying the size-dependent dynamic behaviors of micro-structures is crucial for optimal manufacturing and design.

Carbon-based materials, such as carbon nanotubes (CNTs) and graphene platelets (GPLs), are widely recognized for their superior properties and have been used as nanofillers to produce composite materials with exceptional characteristics. GPLs, in particular, have been selected as the reinforcing phase in composites due to their enhanced properties, including a higher Young’s modulus, improved stiffness, outstanding tensile strength, and excellent thermal conductivity [3]. For instance, novel GPL-reinforced polymers have shown significant potential for various engineering applications [4]. Recently, several studies have focused on the mechanical behavior of GPL-reinforced composites (GPLRC). Rafiee et al. [5,6] experimentally examined the buckling load of GPLRC beams and conducted an extensive study on the physical properties of epoxy-based composites reinforced with either GPLs or CNTs. The numerical results demonstrated the advantages of GPLRC over CNT-reinforced composites (CNTRC) [3–4].

A brief review of recent studies on the vibration analysis of cylindrical structures, particularly those made of functionally graded graphene platelets reinforced composites (FG-GPLRC), is presented. Gao et al. [7] investigated the sub-harmonic resonance of a composite cylindrical shell subjected to aerodynamic loading using Donnell's nonlinear shell theory. Jafari et al. [8] conducted a nonlinear vibration analysis of simply-supported FG cylindrical shells with piezoelectric layers, assuming Donnell's nonlinear theory. Hosseini-Hashmi et al. [9] analyzed the vibration of FG viscoelastic cylindrical panels under various boundary conditions using the first-order shear deformation theory (FSDT) in conjunction with Sanders’ theory, employing the state-space technique. The natural frequencies of reinforced curved panels were obtained using a 3D mesh-free procedure by Soltanimaleki et al. [10]. Shen et al. [11] explored the nonlinear vibration behavior of GPLRC shells in a thermal medium, based on third-order shear deformation theory (TSDT) and von Kármán-type relations. Dong et al. [12] provided an analytical solution for the free vibration analysis of rotating GPLRC cylindrical shells. Niu et al. [13] developed the Chebyshev-Ritz method, considering cantilever boundary conditions and FSDT, to determine the natural frequencies of rotating, pre-twisted composite cylindrical shells reinforced with FG-GPLs, which were validated using ANSYS software. Arani et al. [14] investigated the natural frequencies and dynamic responses of FG-CNTRC cylindrical panels using TSDT, employing the differential quadrature method (DQM) and Newmark method. Barati et al. [15] studied the natural frequencies of GPL-reinforced shells using the first-order shell model, considering various GPL distributions along the thickness. Bahaadini et al. [16] examined the vibration of FG porous truncated conical shells reinforced with GPLs, applying DQM under different boundary conditions. Wang et al. [17] obtained the nonlinear natural frequencies of porous GPLRC cylindrical shells with the aid of the multiple timescale method (MSM). Wu et al. [18] analyzed the nonlinear response of forced vibration in FG-CNT reinforced composite cylindrical shells, employing Volmir’s assumption and the incremental harmonic balance method (IHBM). Yadav et al. [19] investigated the nonlinear forced vibration equations of sandwich cylindrical shells with cellular cores, utilizing IHBM and higher-order shear deformation theory. Teng [20] studied the spinning-induced internal resonance of simply-supported cylindrical shells. Dong et al. [21] examined the multi-mode nonlinear oscillation of cylindrical shells and derived amplitude–frequency curves using IHBM. Wang et al. [22] used the Rayleigh–Ritz method and temperature-dependent material properties to investigate the free and forced vibration characteristics of cylindrical shells with arbitrary boundary conditions in a thermal field, utilizing the Spectral-Geometry Method. Zhang and Shi [23] applied MSM and the Galerkin principle to examine the forced vibration behavior and nonlinear primary resonance response of axially moving FG cylindrical shells in a thermal medium.

Using shear deformation theory (SDT) with von Karman strains, Ali and Hasan [24] investigated the nonlinear dynamic stability characteristics and buckling loads of functionally graded material (FGM) toroidal shell panels subjected to axial constant velocity. Their analysis employed Galerkin's technique, the fourth-order Runge–Kutta method, and the Budiansky–Roth criterion. In a subsequent study [25], they examined the vibration behavior of FG toroidal shells with damping, utilizing SDT based on Stein and McElman's assumption and von Karman-type nonlinearity. The study obtained frequency-amplitude relationships and the nonlinear-to-linear frequency ratio. Furthermore, Ali and Hasan [26] developed a robust analytical method to assess the nonlinear dynamic stability of an imperfect plate reinforced with carbon nanotubes (CNTs) under axial force, considering the damping effect, SDT, and nonlinear strains based on the von Karman model. Hasan and Ali [27] introduced an analytical model for the forced vibration of graphene-reinforced composite (GRC) cylindrical shells with viscous damping in a thermal medium and various boundary conditions. The differential equations were derived using SDT with von Kármán-type strains and solved using the multiple scales method to obtain the nonlinear forced vibration frequency responses.

Non-classical elasticity theories, such as strain gradient theory (SGT) and modified couple stress theory (MCST), have been proposed to examine the size-dependent behavior of micro-structures. Researchers have developed models of microstructures using MCST and SGT to analyze size-dependent vibrational responses of microplates and microshells. Beni et al. [28] studied the size-dependent vibration of functionally graded (FG) cylindrical shells using a shear deformation model and MCST. Gholami et al. [29] investigated the dynamic behavior of micro-/nano-shells through strain gradient formulations and MCST. Tohidi et al. [30] explored the dynamic stability of FG-CNTRC microshells subjected to harmonic, non-uniform temperature distributions. Veysi and Shabani [31] derived frequency responses for thick microshells based on MCST, employing the multiple scales method. Rezavi et al. [32] developed governing equations for the electro-mechanical vibration of cylindrical nanoshells made of FG piezoelectric material using MCST. Hasrati et al. [33] proposed a novel numerical solution to describe the nonlinear free and forced oscillations of cylindrical shells using FSDT and nonlinear geometric strains. Wang et al. [17] conducted a vibration analysis of cylindrical shells reinforced with GPLs using Donnell’s nonlinear shell theory. Anvari et al. [34] studied the natural frequencies of cylindrical sandwich micropanels with GPLR layers on a Winkler substrate in a thermal medium, based on MCST, using Navier's method. Bidzard et al. [35] employed the harmonic balance method and a size-dependent finite element model to obtain the nonlinear frequencies of multilayer FG-GPLRC toroidal microshells on a nonlinear elastic foundation in a thermal environment, considering FSDT and von Karman assumptions. Mosayyebi et al. [36] investigated the size-dependent vibration behaviors of viscoelastic sandwich microplates with a GPLRC core and two piezoelectric smart sensor/actuator face layers, using MCST. These sandwich piezoelectric microplates were embedded in a viscoelastic medium and exposed to an electromagnetic field. Yin and Fang [37] obtained the natural frequencies and dynamic responses of spinning FG-GPLRC microplates using MCST and FSDT, employing the state-space technique and fourth-order Runge–Kutta method. Ma et al. [38] investigated the bending response of GPLR cylindrical microcapsules under a moving load using MCST, applying the Laplace transform approach and the double Fourier series method.

Using isogeometric analysis (IGA), Hung et al. [39] obtained the bending responses, critical buckling loads, and natural frequencies of functionally graded (FG) periodic minimal surfaces microplates, based on higher-order shear deformation theory (HSDT) and modified strain gradient theory (MSGT). In a similar study, they applied MSGT to perform free vibration and buckling analyses of porous metal foam microplates using refined HSDT and IGA [40], considering three different porosity patterns. By combining MSGT with IGA, Hung et al. [41] determined the size-dependent deflection and natural frequency of CNT-reinforced magneto-electro-elastic (MEE) microplates, employing the refined HSDT with four variables. They also studied the size-dependent thermal vibration and buckling behavior of FG-MEE microplates in a thermal medium, using MSGT in conjunction with generalized HSDT [42]. Additionally, Hung et al. [43,44] investigated the free vibration characteristics of FG porous smart magneto-electro-elastic plates and honeycomb multi-layered microplates with the assistance of IGA.

A review of the literature reveals that no studies have yet explored the nonlinear primary resonance of size-dependent FG-GPLRC cylindrical microshells in a thermal environment. The temperature field and the size-dependent characteristics of the microshell are expected to significantly influence the vibration behavior of FG-GPLRC cylindrical shells. Inspired by the existing literature, this research aims to investigate the size-dependent nonlinear primary resonance characteristics of FG-GPLRC circular cylindrical microshells in a thermal medium. After deriving the equations of motion using Hamilton’s principle, the Galerkin method and the multiple timescale method (MSM) are employed to solve them analytically, yielding the nonlinear frequency-amplitude curve for primary resonance.

## 2 Mathematical modeling

### 2.1 GPL reinforced composite

**Fig 1:**
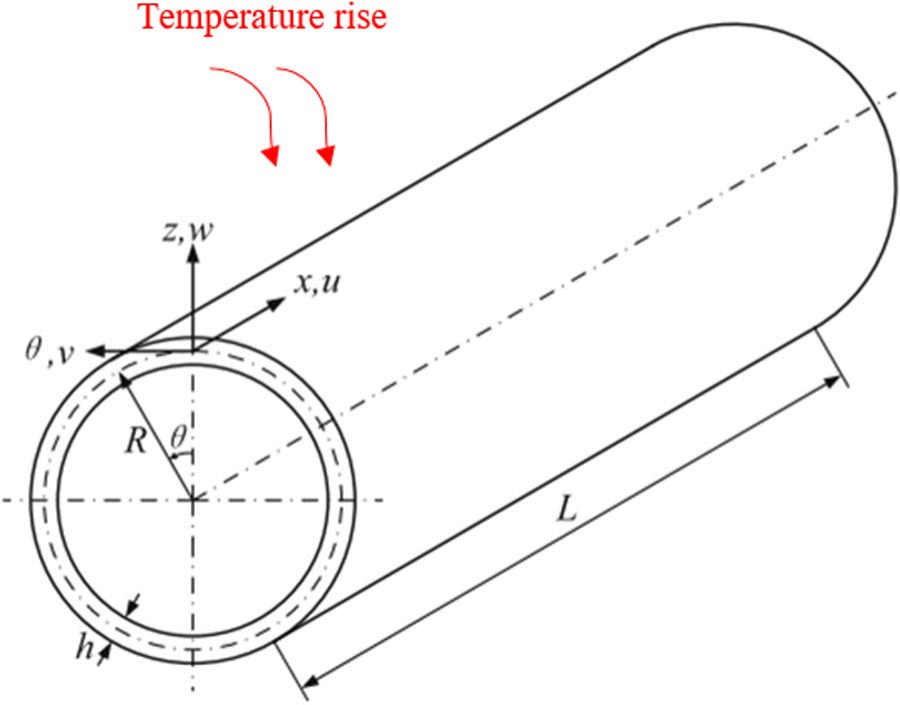
Schematic of cylindrical shell in thermal medium.

**Fig 2:**
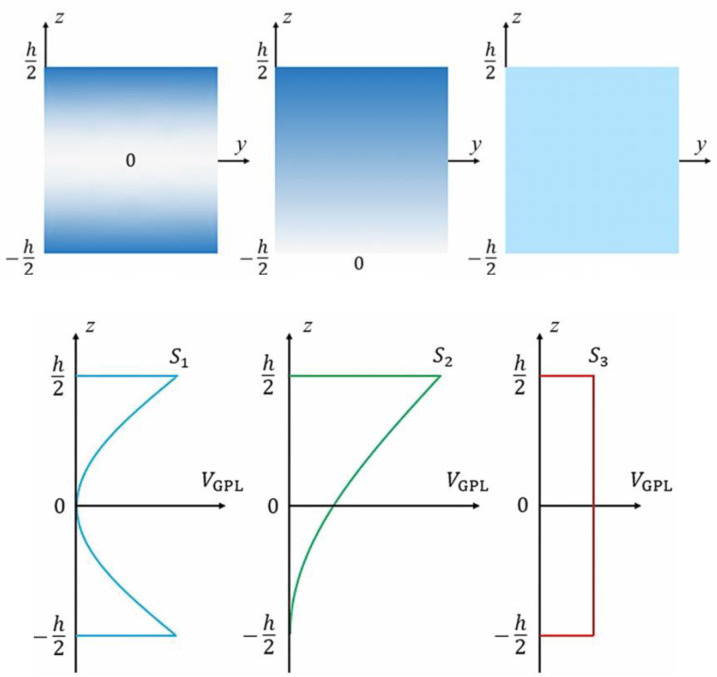
Distribution of different GPL patterns.


Fig 1 presents a GPLRC cylindrical micro shell in a thermal environment. As shown in the Fig 1; h, L and r, represent the thickness, length and radius of the cylindrical shell, respectively. Also, for GPL dispersion patterns the varied GPLs volume fraction along the core thickness, as depicted in Fig 2 is expressed as [12]:

$$\left\{\begin{array}{c} g_{1}(z)\\ g_{2}(z)\\ g_{3}(z) \end{array}\}=[\begin{array}{c} 1\\ 1-cos\left(\frac{\pi z}{h}\right)\\ 1-cos\left(\frac{\pi z}{2h}+\frac{\pi }{4}\right) \end{array}\right]$$ (1)

Where $g_{i}\left(z\right)\;\left(i=1,2,3\right)$ are the shape functions of GPL. Also, the function of GPL volume fraction is $V_{gpl}=S_{i}g_{i}\left(z\right)$, in which $S_{i}$ is the peak values of GPLs volume fraction and equals to: ${\text{S}}_{i}=\frac{1}{\int_{-h/2}^{h/2}g_{i}\left(z\right)dz}\times \frac{h\;{\text{g}}_{GPL}}{{\text{g}}_{GPL}+\left(1-{\text{W}}_{GPL}\right)\rho_{GPL}/\rho_{M}}$; in which ${\text{g}}_{GPL}$ is the weight fraction of GPLs. $\rho_{GPL}$ and $\rho_{M}$ represent the mass densities of GPL and matrix, respectively.

Young’s modulus of the GPL reinforced core can be calculated by the Halpin-Tsai micromechanics algorithm [6], yielding:


$$E_{nc}=E_{M}\left[\frac{3}{8}\left(\frac{1+2\xi_{L}\eta_{L}V_{GPL}}{1-\eta_{L}V_{GPL}}\right)+\frac{5}{8}\left(\frac{1+2\xi_{B}\eta_{B}V_{GPL}}{1-\eta_{B}V_{GPL}}\right)\right]$$ (2)

In which:


$$\;\xi_{L}=\frac{l_{GPL}}{t_{GPL}}\;,\;\xi_{B}=\frac{b_{GPL}}{t_{GPL}}\;,\;\eta_{L}=\frac{E_{GPL}/E_{M}-1}{E_{GPL}/E_{M}+2\xi_{L}}\;,\;\eta_{B}=\frac{E_{GPL}/E_{M}-1}{E_{GPL}/E_{M}+2\xi_{B}}\;,\;$$ (3)

Where $l_{GPL}$,$\;\;\;\;\;\;\;b_{GPL}$, and $t_{GPL}$ are the GPLs’ average length, width, and thickness, respectively. E_M_ and $E_{GPL}\;$are elastic moduli of the matrix and GPL materials of core, respectively. In addition, on the basis of the rule of mixture, the Poisson’s ratio, thermal expansion and density of the GPLs reinforced matrix of core are defined as [34]:

$\upsilon_{nc}=\upsilon_{GPL}V_{GPL}+\upsilon_{M}\left(1-V_{GPL}\right)$ $\alpha_{nc}=\alpha_{GPL}V_{GPL}+\alpha_{M}\left(1-V_{GPL}\right)$


$$\rho_{nc}=\rho_{GPL}V_{GPL}+\rho_{M}\left(1-V_{GPL}\right)$$ (4)

In which $\upsilon_{m}$, $\upsilon_{GPL}$ are the Poisson's ratios of the matrix and GPLs nanofillers, respectively. Also $\alpha_{M}$ and $\alpha_{GPL}$ are the thermal expansion of the matrix and GPLs of core, respectively. The coefficient of thermal conductivity for the GPLs’ reinforced material is [45]:


$$k_{nc}=k_{M}\left\{1+\frac{V_{GPL}}{3}\left[\left(\frac{2}{H+1/\left(\frac{k_{x}}{k_{M}}-1\right)}+\frac{1}{\left(1-H\right)/2+1/\left(\frac{k_{z}}{k_{M}}-1\right)}\right)+1\right]\right\}$$ (5)

In Eq. (5):


$$H=\frac{Ln\left[\left(\xi_{L}+\sqrt{{\xi_{L}}^{2}-1}\right)\xi_{L}\right]}{\sqrt{{\left({\xi_{L}}^{2}-1\right)}^{3}}}-\frac{1}{{\xi_{L}}^{2}-1}$$ (6)

Besides, in the Eq. (5), $k_{z}$ and $k_{x}$ are the GPLs’ thermal conductivity coefficients, equals to:


$$k_{x}=\frac{k_{GPL}}{1+2R_{K}k_{GPL}/l_{GPL}}\;;\;k_{z}=\frac{k_{GPL}}{1+2R_{K}k_{GPL}/t_{GPL}}\;$$ (7)

Where $k_{GPL}$ and R_k_ parameters reported in Ref. [45].

The thermal boundary conditions at the inner and outer surface of microshell are: T _(−h/2)_ = T_i_ and T _(h/2)_ = T_o_, and the temperature variation along the shell thickness can be obtained by solving the one-dimensional Fourier law of heat conduction $-\frac{d}{dz}\left(K\left(z\right)\frac{dT\left(z\right)}{dz}\right)=0$. So, the temperature distribution of microshell equals to: T (z) = $\hat{T}+\;\;\;\Delta \;\;\;T$, where $\hat{T}$ denotes the early temperature at the free stress condition, and  Δ T is the temperature variation. Through the thickness distribution of the temperature are equals to [45]:


$$T\left(z\right)=\frac{T_{0}-T_{i}}{\int_{-h/2}^{h/2}\left[d\eta /k_{nc}\left(\eta \right)\right]}\underset{-h/2}{\overset{z}{\int }}\;\left[d\eta /k_{nc}\left(\eta \right)\right]+\hat{T}$$ (8)

where k_nc_(η) is the coefficient of thermal conductivity of the GPLRC.

### 2.2 Hamilton principles using MCST

An orthogonal coordinate system (x_1_, x_2_, z) is adopted. u_0_, v_0_, and w_0_ are components of displacements of the middle surface (z=0) in the x, θ, and z-directions, respectively. Consistent with the shear deformation assumptions, the displacement field stated as [9]:


$$u_{1}\left(x_{1},x_{2},z,t\right)=u\left(x_{1},x_{2},t\right)+z\phi_{1}\left(x_{1},x_{2},t\right)$$

$$v_{1}\left(x_{1},x_{2},z,t\right)=v\left(x_{1},x_{2},t\right)+z\phi_{2}\left(x_{1},x_{2},t\right)$$

$$w_{1}\left(x_{1},x_{2},z,t\right)=w\left(x_{1},x_{2},t\right)$$ (9)

In which (u, v, w) are the mid-surface displacements at coordinate ($x_{1},x_{2}$, 0) of circular cylindrical shell and ($\phi_{1}$, $\phi_{2}$) are the normal rotations. Considering the above displacement field and geometrically nonlinear relations, the membrane strains, curvatures and the transverse shear strains are as follows [17]:


$$\varepsilon_{1}=\frac{\partial u}{\partial x_{1}}+\frac{1}{2}{\left(\frac{\partial w}{\partial x_{1}}\right)}^{2}\;$$

$$\varepsilon_{2}=\frac{1}{R}\left(\frac{\partial v}{\partial x_{2}}+w\right)+\frac{1}{2}{\left(\frac{1}{R}\frac{\partial w}{\partial x_{2}}\right)}^{2}$$

$$\varepsilon_{12}=\frac{\partial v}{\partial x_{1}}+\frac{1}{R}\frac{\partial u}{\partial x_{2}}+\frac{1}{R}\frac{\partial w}{\partial x_{1}}\frac{\partial w}{\partial x_{2}}$$

$$\varepsilon_{13}=\frac{\partial w}{\partial x_{1}}+\phi_{1}\;;\;\varepsilon_{23}=\frac{1}{R}\frac{\partial w}{\partial x_{2}}+\phi_{2}$$

$$\kappa_{1}=\frac{\partial \phi_{1}}{\partial x_{1}}\;;\;\kappa_{2}=\frac{1}{R}\frac{\partial \phi_{2}}{\partial x_{2}}\;;\kappa_{12}=\frac{\partial \phi_{2}}{\partial x_{1}}+\frac{1}{R}\frac{\partial \phi_{1}}{\partial x_{2}}$$ (10)

Based on the MCST and considering length scale parameter, the virtual strain energy of circular cylindrical micro shell equals to [37]:


$$\delta U_{s}=\frac{1}{2}\underset{s}{\overset{\prime }{\int }}\;\underset{-h/2}{\overset{h/2}{\int }}\;\left(\sigma_{ij}\delta \varepsilon_{ij}+m_{ij}\delta \chi_{ij}\right)R\;dx_{1}dx_{2}dz$$ (11)

In which the stress components $\sigma_{ij}$ are equals to:


$${\left\{\sigma_{11}\;\sigma_{22}\;\sigma_{12}\;\sigma_{23}\;\sigma_{13}\right\}}^{T}=\left[\;\;\;\Gamma \;\;\;\left(z\right)\mathrm{\backslash rightleft}({\left\{\varepsilon_{11}\;\varepsilon_{22}\;\varepsilon_{12}\;\varepsilon_{23}\;\varepsilon_{13}\right\}}^{T}-\Delta T\left(z\right){\left\{\alpha_{1}\;\alpha_{2}\;0\;0\;0\right\}}^{T}\right)$$ (12)

In which, [ Γ (z)] are the matrices of stiffness coefficients, are defined according to:


$${\;\;\;\Gamma \;\;\;}_{11}={\;\;\;\Gamma \;\;\;}_{22}=\frac{E\left(z\right)}{1-\upsilon {\left(z\right)}^{2}}\;,\;{\;\;\;\Gamma \;\;\;}_{12}=\frac{\upsilon \left(z\right)E\left(z\right)}{1-\upsilon {\left(z\right)}^{2}}\;,{\;\;\;\Gamma \;\;\;}_{44}={\;\;\;\Gamma \;\;\;}_{55}={\;\;\;\Gamma \;\;\;}_{66}=\frac{E\left(z\right)}{2\left(1+\upsilon \left(z\right)\right)}$$ (13)

The thermal expansion coefficients ($\alpha_{1},\alpha_{2}$) related to the (x, θ) directions supposed to be equal $\alpha_{1}$, $\alpha_{2}=\alpha_{eff}$. Furthermore, the higher-order stress $m_{ij}$ are the functions of $\chi_{ij}$ (curvature tensors), based on the next formula [37]:


$$m_{ij}=2l^{2}\mu (z)\chi_{ij};\;(\;i,j=1,2,3)$$ (14)

Where μ(z) is the Lamé constant of material, and l is scale parameter of material.

Assuming the geometric relation (1+z/R=1) in a thin shell, and with consideration of Eq. (8):


$$\chi_{11}=\frac{1}{2R}\left(\frac{\partial^{2}w}{\partial x_{1}\partial x_{2}}-\frac{\partial v}{{\partial x}_{1}}\right)-\frac{1}{2}\frac{\partial \phi_{2}}{{\partial x}_{1}}$$

$$\chi_{22}=\frac{1}{2R}\left(\frac{\partial \phi_{1}}{{\partial x}_{2}}-\frac{\partial^{2}w}{\partial x_{1}\partial x_{2}}+\frac{\partial v}{{\partial x}_{1}}+z\frac{\partial \phi_{2}}{{\partial x}_{1}}-\frac{1}{R}\frac{\partial u}{{\partial x}_{2}}\right)$$

$$\chi_{33}=\frac{1}{2}\left[\frac{\partial \phi_{2}}{{\partial x}_{1}}-\frac{1}{R}\left(\frac{\partial \phi_{1}}{{\partial x}_{2}}-\frac{1}{R}\frac{\partial u}{{\partial x}_{2}}\right)\right]$$

$$\chi_{12}=\chi_{21}=\frac{1}{4}\left[\frac{\partial \phi_{1}}{{\partial x}_{1}}-\frac{\partial^{2}w}{\partial x_{1}^{2}}-\frac{1}{R}\frac{\partial \phi_{2}}{{\partial x}_{2}}+\frac{1}{R^{2}}\left(\frac{\partial^{2}w}{\partial x_{2}^{2}}-\frac{\partial v}{{\partial x}_{2}}\right)\right]$$

$$\chi_{13}=\chi_{31}=\frac{1}{4}\left[\frac{\partial^{2}v}{\partial x_{1}^{2}}+z\frac{\partial^{2}\phi_{2}}{\partial x_{1}^{2}}+\frac{1}{R}\left(-\frac{\partial^{2}u}{\partial x_{1}\partial x_{2}}-z\frac{\partial^{2}\phi_{1}}{\partial x_{1}\partial x_{2}}\right)-\frac{1}{R}\phi_{2}+\frac{1}{R^{2}}\left(v-\frac{\partial w}{{\partial x}_{2}}\right)\right]$$

$$\chi_{23}=\chi_{32}=\frac{1}{4}\left[\frac{1}{R}\left(\frac{\partial^{2}v}{\partial x_{1}\partial x_{2}}+z\frac{\partial^{2}\phi_{2}}{\partial x_{1}\partial x_{2}}-\phi_{1}+\frac{\partial w}{{\partial x}_{1}}\right)-\frac{1}{R^{2}}\left(\frac{\partial^{2}u}{\partial x_{2}^{2}}+z\frac{\partial^{2}\phi_{1}}{\partial x_{2}^{2}}\right)\right]$$ (15)

The virtual potential energy due to the applied distributed load *f = f*_*0*_ *cos* Ω*t* can be given by:


$$\delta W_{q}=\int_{S}q_{z}(x_{1},x_{2},t)\delta wRdx_{1}dx_{2}$$ (16)

Where: *qz (*$x_{1},x_{2}$*, z) = f*_*0*_ *cos Ωt × sin(mπx*_*1*_*/L) ×cos (nx*_*2*_).

For the microshell, the virtual kinetic energy of the microshell can be given by:


$$\delta K=\int_{S}\int_{-\frac{h}{2}}^{\frac{h}{2}}\rho_{eff}(z)\left[\left(\dot{u}+z{\dot{\phi }}_{1}\right)\left(\delta \dot{u}+z{\delta \dot{\phi }}_{1}\right)+\left(\dot{v}+z{\dot{\phi }}_{2}\right)\left(\delta \dot{v}+z{\delta \dot{\phi }}_{2}\right)+\dot{w}\delta \dot{w}\right]Rdx_{1}dx_{2}dz$$ (17)

Applying the Rayleigh dissipation function [46], the work done on the system by the viscous damping force, equals to (c is the viscous friction coefficient):


$$\delta W_{d}=\int_{S}\int_{-\frac{h}{2}}^{\frac{h}{2}}c\left[\left({\dot{u}}^{2}+{\dot{v}}^{2}+{\dot{w}}^{2}\right)\right]dx_{1}dx_{2}dz$$ (18)

Governing equations of the system, can be obtained by means of Hamilton’s principle, as follow:


$$\delta \int_{t_{1}}^{t_{2}}(W_{q}+W_{d}+K-U_{s})dt$$ (19)

By substituting Eqs. (11), (16), (17), and (18) into Eq. (19) and letting the coefficients in front of δu, δv, δw, δ$\phi_{1}$, and δ$\phi_{2}$ to be zero, respectively; the governing equations in the PDE form are:


$$\delta u\;:\;\frac{\partial N_{1}}{\partial x_{1}}+\frac{1}{R}\frac{\partial N_{12}}{\partial x_{2}}-\frac{1}{2R^{2}}\frac{\partial Y_{22}}{\partial x_{2}}+\frac{1}{2R^{2}}\frac{\partial Y_{33}}{\partial x_{2}}+\frac{1}{2R}\frac{\partial^{2}Y_{13}}{\partial x_{1}\partial x_{2}}+\frac{1}{2R^{2}}\frac{\partial^{2}Y_{23}}{\partial x_{2}^{2}}=I_{0}\ddot{u}+I_{1}{\ddot{\phi }}_{1}+c\dot{u}$$ (20a)

$$\delta v\;:\;\frac{1}{R}\frac{\partial N_{2}}{\partial x_{2}}+\frac{\partial N_{12}}{\partial x_{1}}+\frac{1}{R}Q_{2}-\frac{1}{2R}\frac{\partial Y_{11}}{\partial x_{1}}+\frac{1}{2R}\frac{\partial Y_{22}}{\partial x_{1}}-\frac{1}{2R^{2}}\frac{\partial Y_{12}}{\partial x_{2}}-\frac{1}{2}\frac{\partial^{2}Y_{13}}{\partial x_{1}^{2}}-\frac{1}{2R^{2}}Y_{13}-\frac{1}{2R}\frac{\partial^{2}Y_{23}}{\partial x_{1}\partial x_{2}}=I_{0}\ddot{v}+I_{1}{\ddot{\phi }}_{2}++c\dot{v}$$ (20b)

$$\begin{array}{c} \delta w:\;\frac{\partial Q_{2}}{\partial x_{2}}+\frac{\partial Q_{1}}{\partial x_{1}}-\frac{1}{R}N_{2}+\eta \left(u,v,w,\phi_{1},\phi_{2}\right)+q_{z}-\frac{1}{2R}\frac{\partial^{2}Y_{11}}{\partial x_{1}\partial x_{2}}+\frac{1}{2R}\frac{\partial^{2}Y_{22}}{\partial x_{1}\partial x_{2}}-\frac{1}{2R^{2}}\frac{\partial^{2}Y_{12}}{\partial x_{2}^{2}}\\ +\frac{1}{2}\frac{\partial^{2}Y_{12}}{\partial x_{1}^{2}}-\frac{1}{2R^{2}}\frac{\partial Y_{13}}{\partial x_{2}}+\frac{1}{2R}\frac{\partial Y_{23}}{\partial x_{1}}=I_{0}\ddot{w}+c\dot{w} \end{array}$$ (20c)

$$\delta \phi_{1}\;:\;\frac{\partial M_{1}}{\partial x_{1}}+\frac{1}{R}\frac{\partial M_{12}}{\partial x_{2}}-Q_{1}+\frac{1}{2R}\frac{\partial Y_{22}}{\partial x_{2}}+\frac{1}{2R}\frac{\partial Y_{33}}{\partial x_{2}}+\frac{1}{2}\frac{\partial Y_{12}}{\partial x_{1}}+\frac{1}{2R}\frac{\partial^{2}T_{13}}{\partial x_{2}^{2}}+\frac{1}{2R}Y_{23}=I_{1}\ddot{u}+I_{2}{\ddot{\phi }}_{1}$$ (20d)

$$\delta \phi_{2}\;:\;\frac{1}{R}\frac{\partial M_{2}}{\partial x_{2}}+\frac{\partial M_{12}}{\partial x_{1}}-Q_{2}-\frac{1}{2}\frac{\partial Y_{11}}{\partial x_{1}}+\frac{1}{2R}\frac{\partial T_{22}}{\partial x_{1}}+\frac{1}{2}\frac{\partial Y_{12}}{\partial x_{2}}-\frac{1}{2}\frac{\partial^{2}T_{13}}{\partial x_{1}^{2}}+\frac{1}{2R}Y_{13}-\frac{1}{2R}\frac{\partial^{2}T_{23}}{\partial x_{1}\partial x_{2}}=I_{1}\ddot{v}+I_{2}{\ddot{\phi }}_{2}$$ (20e)

In which, the mass moments are equals to $I_{i}=\int_{-h/2}^{h/2}\rho (z)(1+z/R)z^{i}dz\;;(i=0,1,2)$.

The expression $\eta \left(u,v,w,\phi_{1},\phi_{2}\right)$ is the nonlinear term due to nonlinear strains, represent as:


$$\eta \left(u,v,w,\phi_{1},\phi_{2}\right)=\frac{\partial }{\partial x_{1}}\left(N_{1}\frac{\partial w}{\partial x_{1}}\right)+\frac{1}{R^{2}}\frac{\partial }{\partial x_{2}}\left(N_{2}\frac{\partial w}{\partial x_{2}}\right)+\frac{1}{R}\frac{\partial }{\partial x_{2}}\left(N_{12}\frac{\partial w}{\partial x_{1}}\right)+\frac{1}{R}\frac{\partial }{\partial x_{1}}\left(N_{12}\frac{\partial w}{\partial x_{2}}\right)$$ (21)

The stress resultants ($N_{1}$,$\;N_{2}$, $N_{12}$), ($M_{1}$,$\;M_{2}$, $M_{12}$) and ($Q_{1}$,$\;Q_{2}$) with thermal effects and the couple stress higher-order stress resultants $Y_{ij},T_{ij}$ can also be given as:


$$\left\{\begin{array}{c} N_{1}\\ N_{2}\\ N_{12} \end{array}\}=[\begin{array}{ccc} A_{11} & A_{12} & 0\\ A_{21} & A_{22} & 0\\ 0 & 0 & A_{66} \end{array}]\{\begin{array}{c} \varepsilon_{1}\\ \varepsilon_{2}\\ \varepsilon_{12} \end{array}\}+[\begin{array}{ccc} B_{11} & B_{12} & 0\\ B_{21} & B_{22} & 0\\ 0 & 0 & B_{66} \end{array}]\{\begin{array}{c} \kappa_{1}\\ \kappa_{2}\\ \kappa_{12} \end{array}\}-\{\begin{array}{c} N_{1}^{T}\\ N_{2}^{T}\\ N_{12}^{T} \end{array}\right\}$$

$$\left\{\begin{array}{c} M_{1}\\ M_{2}\\ M_{12} \end{array}\}=[\begin{array}{ccc} B_{11} & B_{12} & 0\\ B_{21} & B_{22} & 0\\ 0 & 0 & B_{66} \end{array}]\{\begin{array}{c} \varepsilon_{1}\\ \varepsilon_{2}\\ \varepsilon_{12} \end{array}\}+[\begin{array}{ccc} D_{11} & D_{12} & 0\\ D_{21} & D_{22} & 0\\ 0 & 0 & D_{66} \end{array}]\{\begin{array}{c} \kappa_{1}\\ \kappa_{2}\\ \kappa_{12} \end{array}\}-\{\begin{array}{c} M_{1}^{T}\\ M_{2}^{T}\\ M_{12}^{T} \end{array}\right\}$$

$$\left\{\begin{array}{c} Q_{1}\\ Q_{2} \end{array}\}=[\begin{array}{cc} E_{44} & 0\\ 0 & E_{55} \end{array}]\{\begin{array}{c} \varepsilon_{13}\\ \varepsilon_{23} \end{array}\right\}$$

$$\left\langle \{\begin{array}{c} N_{1}^{T}\\ N_{2}^{T}\\ N_{12}^{T} \end{array}\},\{\begin{array}{c} M_{1}^{T}\\ M_{2}^{T}\\ M_{12}^{T} \end{array}\}\right\rangle =\int_{-h/2}^{h/2}\left\{\begin{array}{c} Q_{11}(z)\alpha_{1}(z)+Q_{12}{(z)\alpha }_{2}(z)\\ Q_{12}{(z)\alpha }_{1}(z)+Q_{22}(z)\alpha_{2}(z)\\ 0 \end{array}\}\Delta T(z\right)\left\langle 1,z\right\rangle dz$$

$$\left\langle Y_{ij},T_{ij}\right\rangle =\int_{-h/2}^{h/2}m_{ij}\left\langle 1,z\right\rangle dz$$ (22)

In which:


$$\left\{A_{ij},B_{ij},D_{ij}\right\}=\int_{-h/2}^{h/2}Q_{ij}(z)\left\{1,z,z^{2}\right\}dz$$

$$\left\{E_{44},E_{55}\right\}=\int_{-h/2}^{h/2}\left\{Q_{44}(z),Q_{55}(z)\right\}dz$$ (23)

Where:


$$\;Q_{11}=\Gamma_{11},Q_{12}=\frac{\Gamma_{12}}{\left(1+z/R\right)},Q_{21}=\Gamma_{21},Q_{22}=\frac{\Gamma_{22}}{\left(1+z/R\right)}\;$$

$$Q_{44}=\kappa_{1}\Gamma_{44}\;;\;Q_{55}=\kappa_{2}\frac{\Gamma_{55}}{\left(1+z/R\right)};\;Q_{66}=\frac{\Gamma_{66}}{\left(1+z/R\right)}$$ (24)

In which $\kappa_{1},\;\kappa_{2}$ are modified shear correction factors and equals to $\kappa_{j}=\frac{R_{j}^{2}}{d_{j}J_{j}}\;$, where $R_{j},d_{j},J_{j}$ calculated according to Ref [47]. For homogeneous shell, the shear correction factor are equals to $\kappa_{1}=\kappa_{1}=5/6$.

The coupled Eqs. (20a-20e) can be transformed into the displacements field (u, v, w) and rotations ($\phi_{1}$, $\phi_{2}$). Consequently, the equations of motion are as follows:


$$L_{11}u+L_{12}v+L_{13}w+L_{14}\phi_{1}+L_{15}\phi_{2}+l^{2}\left(L_{11}^{l}u+L_{12}^{l}v+L_{13}^{l}w+L_{14}^{l}\phi_{1}+L_{15}^{l}\phi_{2}\right)+L_{16}ww_{1}^{\prime }+L_{17}ww_{2}^{\prime }=I_{0}\ddot{u}+I_{1}{\ddot{\phi }}_{1}+c\dot{u}$$ (25a)

$$L_{21}u+L_{22}v+L_{23}w+L_{24}\phi_{1}+L_{25}\phi_{2}+l^{2}\left(L_{21}^{l}u+L_{22}^{l}v+L_{23}^{l}w+L_{24}^{l}\phi_{1}+L_{25}^{l}\phi_{2}\right)+L_{26}ww_{1}^{\prime }+L_{27}ww_{2}^{\prime }=I_{0}\ddot{v}+I_{1}{\ddot{\phi }}_{2}+c\dot{v}$$ (25b)

$$\begin{array}{c} L_{31}u+L_{32}v+L_{33}w+L_{34}\phi_{1}+L_{35}\phi_{2}{+l}^{2}\left(L_{31}^{l}u+L_{32}^{l}v+L_{33}^{l}w+L_{34}^{l}\phi_{1}+L_{35}^{l}\phi_{2}\right)+L_{36}ww_{1}^{\prime }\\ +L_{37}ww_{2}^{\prime }+\eta \left(u,v,w,\phi_{1},\phi_{2}\right)+q_{z}=I_{0}\ddot{w}+c\dot{w} \end{array}$$ (25c)

$$L_{41}u+L_{42}v+L_{43}w+L_{44}\phi_{1}+L_{45}\phi_{2}+l^{2}\left(L_{41}^{l}u+L_{42}^{l}v+L_{43}^{l}w+L_{44}^{l}\phi_{1}+L_{45}^{l}\phi_{2}\right)+L_{46}ww_{1}^{\prime }+L_{47}ww_{2}^{\prime }=I_{1}\ddot{u}+I_{2}{\ddot{\phi }}_{1}$$ (25d)

$$L_{51}u+L_{52}v+L_{53}w+L_{54}\phi_{1}+L_{55}\phi_{2}+l^{2}\left(L_{51}^{l}u+L_{52}^{l}v+L_{53}^{l}w+L_{54}^{l}\phi_{1}+L_{55}^{l}\phi_{2}\right)+L_{56}ww_{1}^{\prime }+L_{57}ww_{2}^{\prime }=I_{1}\ddot{v}+I_{2}{\ddot{\phi }}_{2}$$ (25e)

In which $L_{ij}$ and $L_{ij}^{l}$ are linear operators.

### 2.4 Solution procedure

In this section, an analytical method based on Galerkin method and multiple scale perturbation technique is applied to solve the coupled nonlinear vibration equations corresponding to the simply supported GPL-reinforced cylindrical microshell. In this regard, the mathematical expression of simply supported BCs can present as v = w = M_11_ = $\phi_{2}=0$ at x = 0, L. Taking into account simply supported boundary conditions, the displacement variables can be selected as the next form [34]:


$$u\left(x_{1},x_{2},t\right)=U(t)cos(\lambda_{m}x)\cos\left(n\theta \right)$$

$$v\left(x_{1},x_{2},t\right)=V(t)sin(\lambda_{m}x)\sin\left(n\theta \right)$$

$$w\left(x_{1},x_{2},t\right)=W(t)\sin(\lambda_{m}x)\cos\left(n\theta \right)$$

$$\phi_{1}\left(x_{1},x_{2},t\right)=\Phi (t)cos(\lambda_{m}x)\cos\left(n\theta \right)$$

$$\phi_{2}\left(x_{1},x_{2},t\right)=\Psi (t)(t)\sin{(\lambda }_{m}x)sin\left(n\theta \right)$$ (26)

Where the time-dependent variables $U_{mn}(t)$, $V_{mn}(t)$, $W_{mn}(t)$,$\;\Phi_{mn}(t)$,$\;\Psi_{mn}(t)$ are coefficients to be obtained in the subsequent section. Also $\lambda_{m}=\frac{m\pi }{L}$, m and (n = 1,2, 3, …) are the half wave numbers corresponding directions x_1_ and x_2_, respectively. For the sake of brevity, the *mn* indexes have not be written in the next relations.

Using Galerkin’s technique and substituting the Eqs. (26) into Eqs. (25a-25e) yields, a set of time-dependent ordinary differential equations (ODEs) can be obtained. Assuming the transverse flexural motion is significant for the thin-walled shell case, an ordinary way is to omit the terms $\ddot{u},{\ddot{\phi }}_{1}\;,\ddot{v},{\ddot{\phi }}_{2}$; which has been stated in literature [48].


$$C_{11}U(t)+C_{12}V(t)+C_{13}W(t)+C_{14}\Phi (t)+C_{15}\Psi (t)+C_{16}{W(t)}^{2}=0$$ (27)

$$C_{21}U(t)+C_{22}V(t)+C_{23}W(t)+C_{24}\Phi (t)+C_{25}\Psi (t)+C_{26}{W(t)}^{2}=0$$ (28)

$$\begin{array}{c} C_{31}U(t)+C_{32}V(t)+C_{33}W(t)+C_{34}\Phi (t)+C_{35}\Psi (t)+C_{36}{W(t)}^{2}+C_{3}^{1}U(t)W(t)\\ +C_{3}^{2}V(t)W(t)+C_{3}^{4}\Phi (t)W(t)+C_{3}^{5}\Psi (t)W(t)+M_{33}\ddot{W}(t)+\delta_{c}\dot{W}(t)=Q(t) \end{array}$$ (29)

$$C_{41}U(t)+C_{42}V(t)+C_{43}W(t)+C_{44}\Phi (t)+C_{45}\Psi (t)+C_{46}{W(t)}^{2}=0$$ (30)

$$C_{51}U(t)+C_{52}V(t)+C_{53}W(t)+C_{54}\Phi (t)+C_{55}\Psi (t)+C_{56}{W(t)}^{2}=0$$ (31)

In which, the coefficients $C_{ij}$ have been defined in Appendix A.

Now, the static condensation procedure based on Volmir’s assumption [49] with an acceptable precision is employed to the Eqs. (27,28,30,31), resulting in the next expression:


$$[\begin{array}{cc} \begin{array}{cc} C_{11} & C_{12}\\ C_{21} & C_{22} \end{array} & \begin{array}{cc} C_{14} & C_{15}\\ C_{24} & C_{25} \end{array}\\ \begin{array}{cc} C_{41} & C_{42}\\ C_{51} & C_{52} \end{array} & \begin{array}{cc} C_{44} & C_{45}\\ C_{54} & C_{55} \end{array} \end{array}][\begin{array}{c} \begin{array}{c} U\\ V \end{array}\\ \begin{array}{c} \Phi \\ \Psi \end{array} \end{array}]=-[\begin{array}{c} \begin{array}{c} C_{13}\\ C_{23} \end{array}\\ \begin{array}{c} C_{43}\\ C_{53} \end{array} \end{array}]W-[\begin{array}{c} \begin{array}{c} C_{16}\\ C_{26} \end{array}\\ \begin{array}{c} C_{46}\\ C_{56} \end{array} \end{array}]W^{2}$$ (32)

By solving Eq. (32), the expressions of U, V, Φ and Ψ can be characterized by W, which are:


$$U=K_{uw}W+K_{uww}W^{2}V=K_{vw}W+K_{vww}W^{2}$$

$$\Phi =K_{xw}W+K_{xww}W^{2}\Psi =K_{yw}W+K_{yww}W^{2}$$ (33)

In which the coefficients of K_αw,_ K_αww_ (α = u, v, x, y) have been supplemented in Appendix B. Then, by replacing Eq. (33) into Eq. (29), the nonlinear vibration equation of circular cylindrical microshell in thermal medium under harmonic excitation can obtained as:


$$\ddot{W}(t)+K_{0}\dot{W}(t)+K_{1}W(t)+K_{2}{W(t)}^{2}+K_{3}{W(t)}^{3}=Q\cos\Omega t$$ (34)

Where:


$$K_{0}=\frac{\delta_{c}}{M_{33}}\;;\;K_{1}=-\frac{1}{M_{33}}\left(C_{31}K_{uw}+C_{32}K_{vw}+C_{34}K_{xw}+C_{35}K_{yw}+C_{33}\right)\;$$

$$K_{2}=-\frac{1}{M_{33}}\left(C_{31}K_{uww}+C_{3}^{1}K_{uw}+C_{32}K_{vww}+C_{3}^{2}K_{vw}+C_{34}K_{xww}+C_{3}^{4}K_{xw}+C_{35}K_{yww}+C_{3}^{5}K_{yw}+C_{36}\right)$$

$$K_{3}=-\frac{1}{M_{33}}\left(C_{3}^{1}K_{uww}+C_{3}^{2}K_{vww}+C_{3}^{4}K_{xww}+C_{3}^{5}K_{yyw}+C_{no}\right)\;;\;Q=\frac{16q}{I_{0}mn\pi^{2}}$$

Besides $\omega =\sqrt{K_{1}}$ equal to linear natural frequency of the microshell, also damping ratio is introduced as $\zeta =K_{0}/(2\sqrt{K_{1}})$.

In this study, the internal resonance was not checked, and just the influence of the chosen mode (m, n) has been considered. Hence, the modal interaction terms can be ignored in Eq. (34), and the nonlinear Eq. (34) can be converted into a simple single-mode equation. Then, the harmonic excitation assumed as Q(t)=QcosΩt, where Ω, Q are the excitation frequency and amplitude of the load corresponding to the anticipated mode (m,n), respectively.

By means of MSM, an approximate analytical solution has been presented and the primary resonance solution considered, in which $\Omega =\omega +\varepsilon^{2}\sigma$; assuming the detuning parameter σ and perturbation parameter ε. From Eq. (35), the perturbation equation obtained as:


$$\ddot{W}(t)+2\varepsilon^{2}\zeta \omega \;\dot{W}(t)+\omega^{2}W(t)+\varepsilon k_{2}{W(t)}^{2}+\varepsilon^{2}k_{3}{W(t)}^{3}=\varepsilon^{2}q\cos\Omega t$$ (35)

The general form of approximate solution of Eq. (36) has been considered as:


$$W(t)=u_{0}\left(T_{0},T_{1},T_{2}\right)+\varepsilon u_{1}\left(T_{0},T_{1},T_{2}\right)+\varepsilon^{2}u_{2}\left(T_{0},T_{1},T_{2}\right)$$ (36)

Where $T_{0}=t,\;T_{1}=\varepsilon t,\;T_{2}=\varepsilon^{2}t$. Replacing Eq. (36) into Eq. (34), and setting the coefficients of ε and $\varepsilon^{2}$ to be equivalent at left and right of the equation, one can obtain:


$$D_{0}^{2}u_{0}+\omega^{2}u_{0}=0$$

$$D_{0}^{2}u_{1}+\omega^{2}u_{1}=-2D_{0}D_{1}u_{0}-k_{2}u_{0}^{2}$$

$$D_{0}^{2}u_{2}+\omega_{mn}^{2}u_{2}=-2D_{0}D_{1}u_{1}-2D_{0}D_{2}u_{0}-D_{1}^{2}u_{0}-2\zeta D_{0}u_{0}-2k_{2}u_{0}u_{1}-k_{3}u_{0}^{3}+q\cos\left(\omega T_{0}+\sigma T_{1}\right)$$ (37)

Solving Eq. (37), then rejecting the secular terms, the solvability condition is:


$$2i\omega \left(A^{\prime }+\zeta A\right)+\left(3k_{3}-\frac{10k_{2}^{2}}{3\omega^{2}}\right)A^{2}\hat{A}-\frac{1}{2}q\exp\left(i\sigma T_{2}\right)=0$$ (38)

In which, parameter A is the vibration amplitude, can be written in the form A = 1/2 $ae^{i\sigma \varsigma }$, where a is the steady-state amplitude.

Separating the real and imaginary portions of Eq. (38), the state equations can be stated as:


$$\dot{a}=\frac{\zeta }{2}a+\frac{q}{2}\sin\xi \;;$$

$$\;\dot{\xi }=\sigma +\frac{3}{8}k_{3}a^{2}-\frac{5}{12}k_{2}^{2}a+\frac{q}{2a}\cos\xi$$ (39)

Where $\xi =\sigma T_{1}-\varsigma$. For the steady-state response, i.e., ($\dot{a}=\dot{\xi }=0$). Hence, the frequency- response of the system for primary resonance state, can be obtained as:


$$\sigma =\left(\frac{9k_{3}\omega^{2}-10k_{2}^{2}}{24\omega^{3}}\right)a^{2}\pm \sqrt{\frac{q^{2}}{4\omega^{2}a^{2}}-\zeta^{2}}$$ (40)

Also, the nonlinear natural frequency of microshell are equals to:


$$\omega_{NL}=\omega -\left(3k_{3}-\frac{10k_{2}^{2}}{3\omega^{2}}\right)\frac{a^{2}}{8\omega }$$ (41)

The stability of steady-state solutions can be found by investigating the singular points of Eq. (39). Presenting disturbance parameters as $a_{s}$ and $\xi_{s}$, one can be written as:


$$a=a_{0}+a_{s}\;;\;\xi =\xi_{0}+\xi_{s}$$ (42)

Where $a_{0}$ and $\xi_{0}$ represent the steady-state solutions. Replacing Eq. (42) into Eq. (39), while recalling the linear terms as and $\xi_{s}$ yields:


$${\dot{a}}_{s}=\frac{\zeta }{2}a_{s}-\xi_{s}(\sigma a_{0}+\frac{3}{8}k_{3}{a_{0}}^{3}-\frac{5}{12}k_{2}^{2}{a_{0}}^{2})\;;$$

$$\;{\dot{\xi }}_{s}=\frac{9}{8}k_{3}a_{0}a_{s}+\frac{\sigma }{a_{0}}a_{s}+\frac{1}{2a_{0}}\zeta a_{0}\xi_{s}$$ (43)

Based on Lyapunov stability theory and Routh-Hurwitz criterion [50], the required and necessary condition for the stability of the steady-state solution is satisfied as follows:


$$\zeta^{2}+4\left(\sigma +\frac{3}{8}k_{3}{a_{0}}^{2}-\frac{5}{12}k_{2}^{2}{a_{0}}^{2}\right)\left(\sigma +\frac{9}{8}k_{3}{a_{0}}^{2}\right)>0$$ (44)

## 3 Results ad discussions

### 3.1 Verification

Natural frequencies parameters $\overset{―}{\omega }=\omega R\sqrt{\frac{\rho }{E}}$ of cylindrical microshell (l=h) have been calculated by MCST, and compared with numerical results from literature for isotropic shells, When L ∕ R = 1, E = 1.06 TPa, ρ = 2300 kg∕m^3^, at Table 1. Acceptable corresponding have been seen among the current study and numerical results obtained by Beni et al. [28] (analytical method) and Gholami [29] (analytical method).

**Table 1:** Comparison of $\overset{―}{\omega }$ for an isotropic shell at different h/R ratios and wave numbers, using MCST.

h/R	n	Present	Beni et al. [28]	Relative Diff (%)	Gholami et al.[29]	Relative Diff (%)
0.1	1	1.135	1.126	0.8	1.132	0.3
	2	1.080	1.069	1.1	1.074	0.6
	3	1.219	1.207	1.0	1.214	0.4
0.2	1	1.571	1.537	2.2	1.538	2.1
	2	1.645	1.590	3.5	1.601	2.7
	3	2.002	1.928	3.8	1.949	2.8

Also as shown in Table 2, to justify the correctness of nonlinear study, the nonlinear-to-linear frequency ratios ($\frac{\omega_{NL}}{\omega_{L}}$) for homogeneous cylindrical shells have been compared with results of Wang et al. [17] (analytical method), Hasrati et al. [33] (GDQ method); with h = 2.55 mm, h/L = 0.006, E = 200 GPa, ρ = 7800 kg∕m^3^ and w_max_/h = 1, the results were in acceptable matching with the literature.

**Table 2:** Validation of $\omega_{NL}/\omega_{L}$ ratio for cylindrical shell for different wave numbers.

Model	Mode		
	n = 2	n = 3	n = 4
Present	1.0014	1.0131	1.0765
Wang et al. [17] (analytical)	1.0009	1.0033	1.0117
Relative Diff (%)	0.05	1.04	6.31
Hasrati et al. [33] (GDQ)	1.0010	1.0072	1.0452
Relative diff (%)	0.04	0.55	2.99

**Fig 3:**
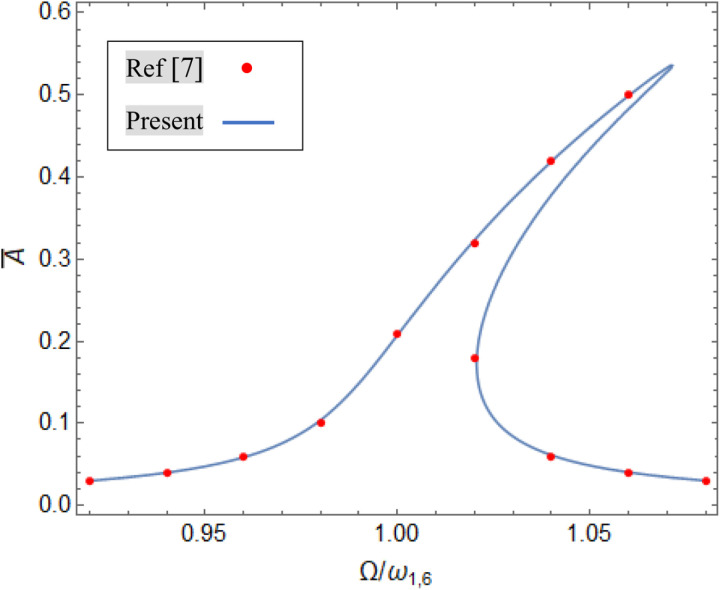
Verification of amplitude-Frequency curve for nonlinear forced vibration with results of Ref [ 7].

Likewise, As shown at Fig 3, the frequency-amplitude curves for primary resonance of forced vibration have been verified by Gao et al. [7] for the Al /Al_2_O_3_ FG cylindrical shell, when: h = 0.25, h/R = 0.01, L/R = 10, (Q = 1KPa, ζ = 0.01, $\overset{―}{A}=\frac{a}{h}$ and FG material power index is p = 0.5.

**Fig 4:**
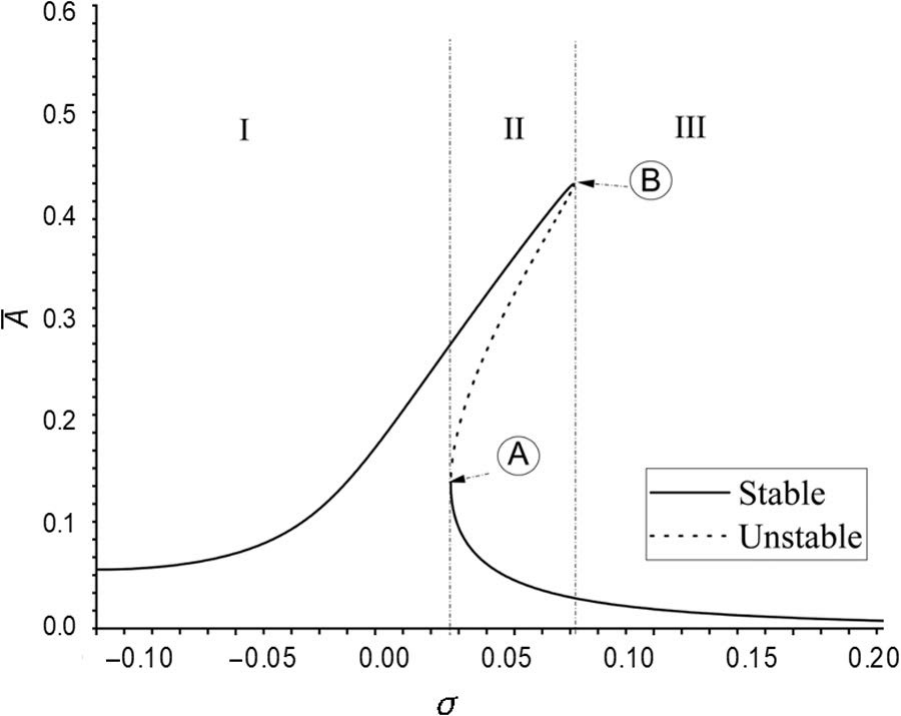
The frequency–amplitude curve of the nonlinear primary.

The frequency–amplitude curve of the primary resonance of system are shown in the Fig 3 and the explanation of its details demonstrated in the Fig 4 by schematic way, in which $\frac{\Omega }{\omega }=1+\sigma$. Three frequency regions can be specified based on the Fig 4. Regions I and III contains one stable curve, while Region II includes two stable and one unstable curve (based on Eq. (44)). The boundary point between the three regions is the bifurcation point [51], therefore the curve includes two bifurcation points, A and B, and hence jump phenomena [51] can be observed

In the Region I, the resonance response amplitude $\overset{―}{A}$ increases as the external excitation frequency σ increases, but it should be pointed out that, when all the diagrams pass the multi-value region, this trend becomes completely inverse (while these trend in the region III is vice versa). In Region II, on the lower curve, $\overset{―}{A}$ increases as σ decreases. In the process of decreasing σ, when passing through the intersection of the dotted line and the solid line (that is, the jump point ‘A’ in Fig 4), $\overset{―}{A}$ will rise from this point to the value corresponding to the σ on the upper solid line, which is called a jump phenomenon. If the peak of the nonlinear resonance response curve shifts to the right, it reflects that the nonlinear type of the resonance curve is the “hardening spring” type.

### 3.2 Parametric analysis for the nonlinear primary resonance

For the next results, the geometrical parameters of the GPLs are t_GPL_ = 2.5 nm, l_GPL_ = 2.5 μm, and b_GPL_ = 1.25 μm. Material properties of main components of the composite have been presented in Table 3.

**Table 3:** Mechanical properties of GPLs and the polymer epoxy [45].

Material	E (GPa)	v	ρ (kg/m^3^)	α (×10^−5^)/K	k (W/mK)	R_k_ (m^2^K/W)
GPL	1010	0.186	1062	2.35	2000	1 × 10 ^−8^
Epoxy	2.85	0.34	1200	8.2	0.2	

In this section, Influences of material length cale parameter, GPLs pattern, GPLs weight fraction, (l/b)_GPL_ and (l/t)_GPL_ on the Nonlinear primary resonance responses of the GPLRC cylindrical microshell have been presented, when ζ=0.02.

**Fig 5:**
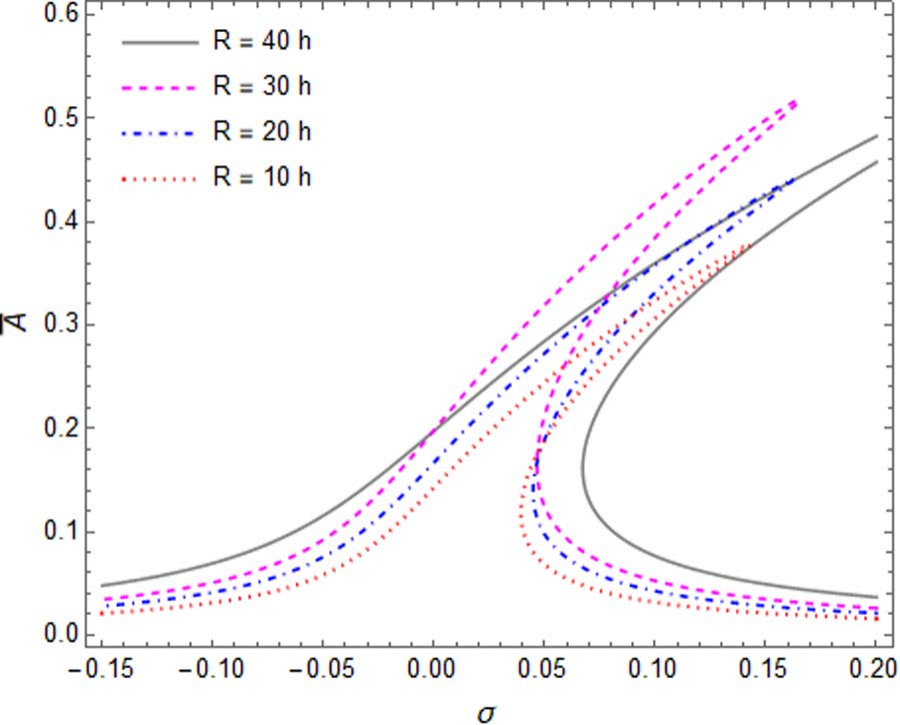
The primary resonance curves of the shell for different value of R/h ratios, when L/R = 4.

Fig 5 demonstrate the effects of the R/h ratio on the primary resonance of nonlinear forced vibration of the cylindrical shell. It can be concluded that by increasing the R/h ratio of the cylindrical shell, if the circumferential wave number of fundamental mode shape remain unchanged, hardening effects decreased; nevertheless, if the fundamental mode wave number (n) rechanged, the hardening effects increased. For example, the circumferential wave number related to the fundamental mode of cylindrical shell change from n = 4 to n = 3, by changing the R/h ratio from R/h = 40 to R/h = 30.

**Fig 6:**
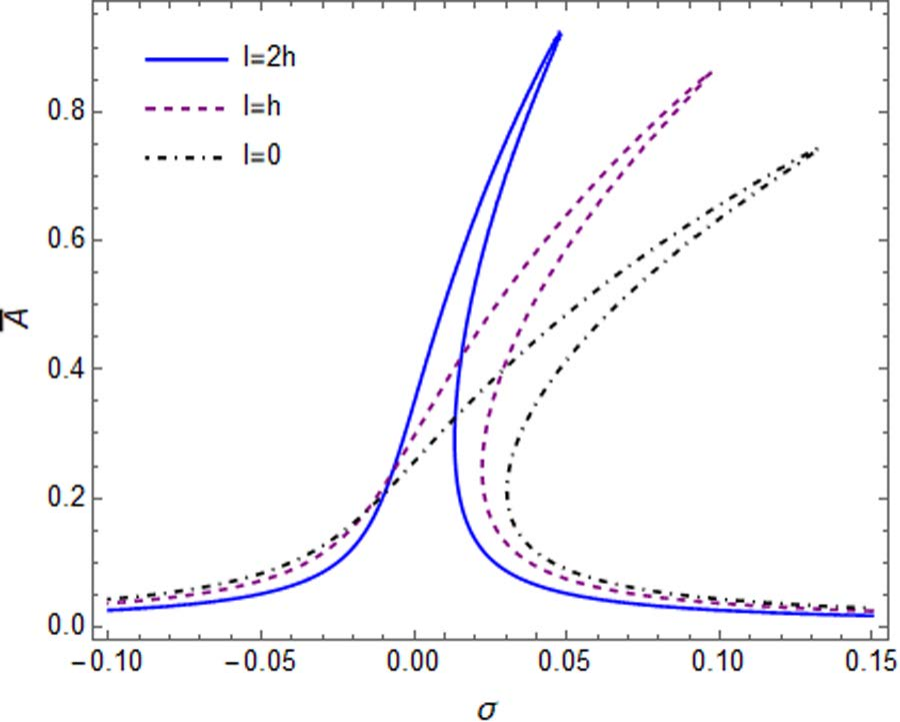
The vibration amplitude ratio versus detuning parameter for different small-scale parameter.

The primary resonances were illustrated in Fig 6 For different values of the small-scale parameter, for the GPL-A reinforced cylindrical microshells with g_GPL_ = 0.1%. Fig 6 indicates that the hardening effect reduced as the scale parameter increased, since the linear stiffnesses of the microshell will increase by rising the l/h parameter. The nonlinear hardening performance increased at smaller l/h ratio microshell as the shell structure becomes stiffer, the geometrical nonlinearity effects, reduced. Besides the multi-valued regions enlarge by decreasing the length scale parameter, so the classical theory is not correct to estimate the bond of unstable responses for microshells.

**Fig 7:**
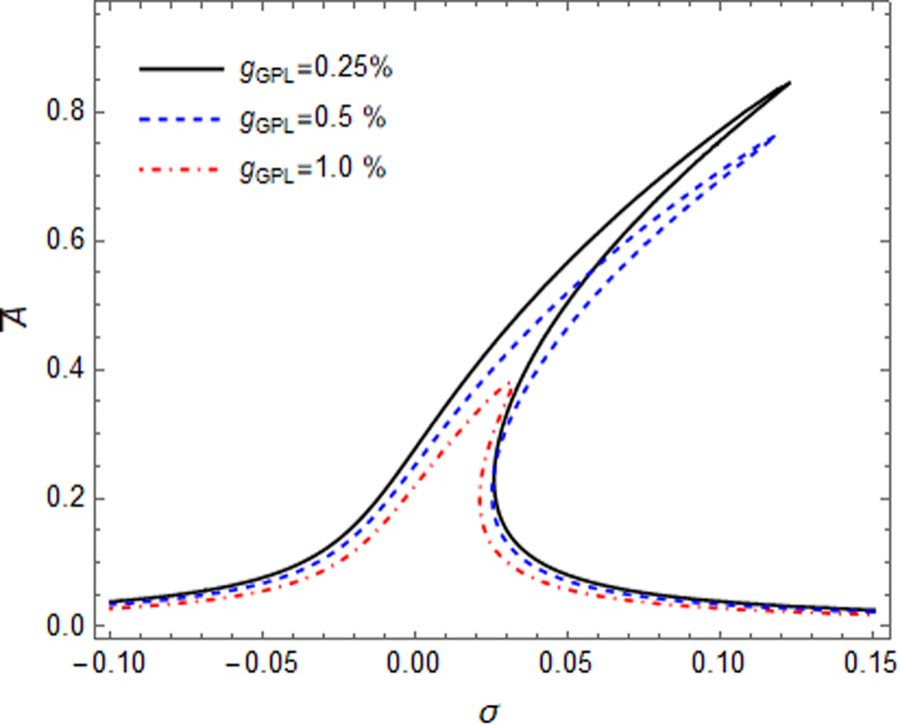
Effect of GPLs weight fraction on primary resonance curves of GPLR shell.

**Fig 8:**
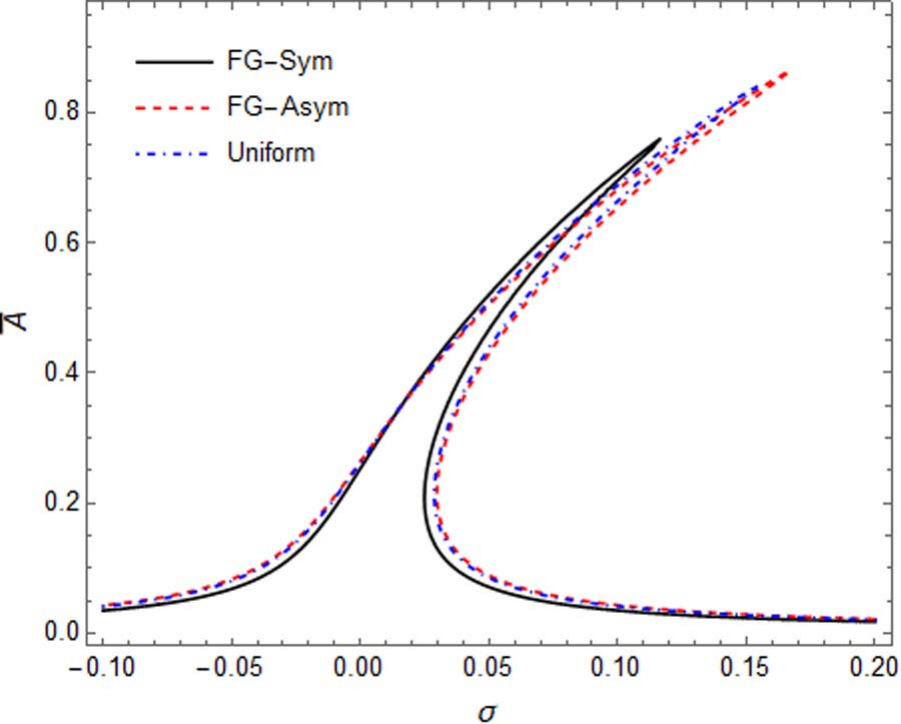
Effect of GPLs patterns on primary resonance curves of GPLR shell.

Impacts of GPL weight fraction and GPL patterns on the primary resonance of GPLRC microshell have been shown in the Fig 7 and Fig 8, respectively. By increasing the parameter g_GPL_, the amplitude of peak point shows a significant drop, and the hardening effect have been decreased, obviously. In Fig 8, the amplitude peak at FG-Sym pattern is the lowest among the three GPLs patterns. This phenomenon exposes that FG-Sym pattern can reach an improved strengthening result arbitrating from the response of the resonance. It can be concluded that by existence of more GPL nearby inner and outer surfaces of the shell, the stiffness increased more.

**Fig 9:**
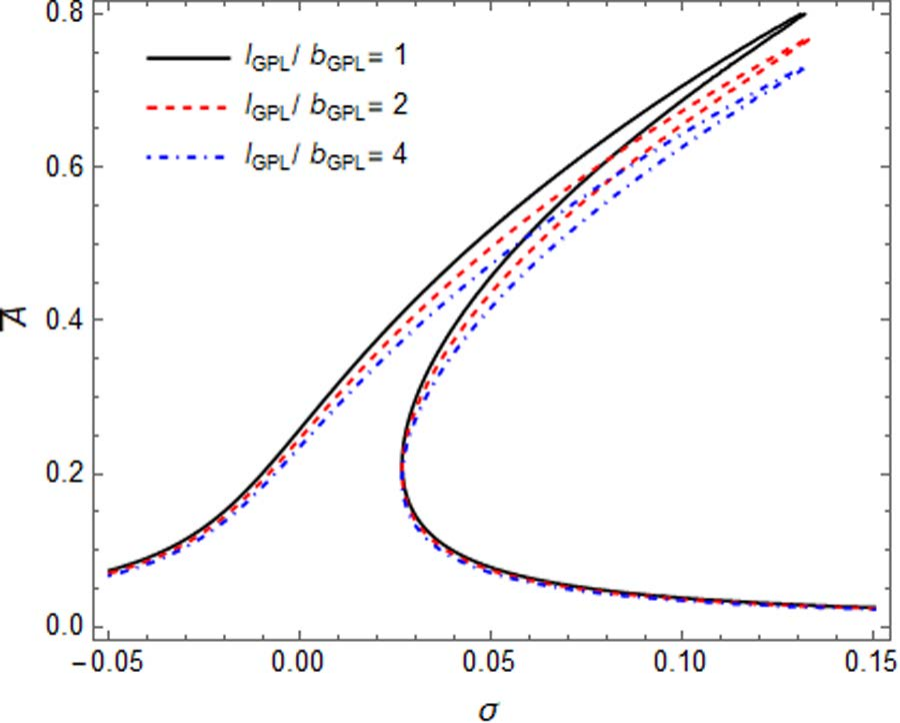
Influence of GPLs size on resonance curves of GPLRC microshell: l_GPL_/b_GPL._

**Fig 10:**
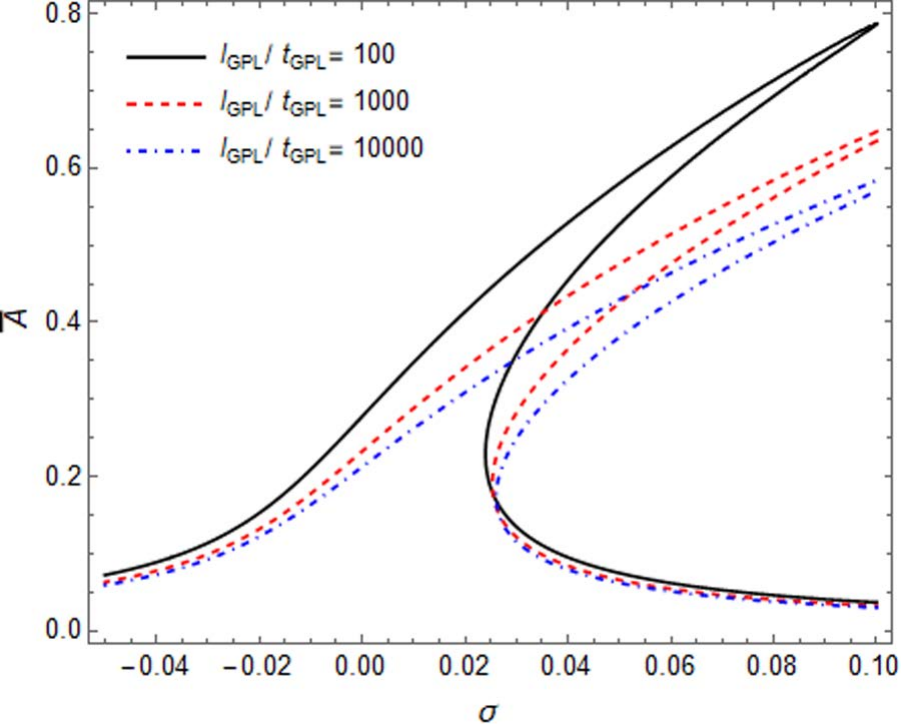
Influence of GPLs size on resonance curves of GPLRC microshell: l_GPL_/t_GPL_ ratio.

The influence of the GPL geometric size on the resonance response of the microshell have been shown in Figs 9, 10 while the l_GPL_ remain fix. In Fig 9, increasing of l_GPL_/b_GPL_ value, leads to an increase in the resonance response amplitude, due to reducing the surface area of GPL (narrowing GPLs) will reduce the linear and nonlinear stiffness of microshell, so “hardening effect” became weak, but the jump point remained almost unchanged. In Fig 10, the resonance response amplitude goes backward and decreased with the increase of l_GPL_/t_GPL_ value. Although the jumping point have small amplitude, due to reducing the thickness of GPLs and increasing the linear and nonlinear stiffness of microshell, and enhancing the “hardening effect” features.

**Fig 11:**
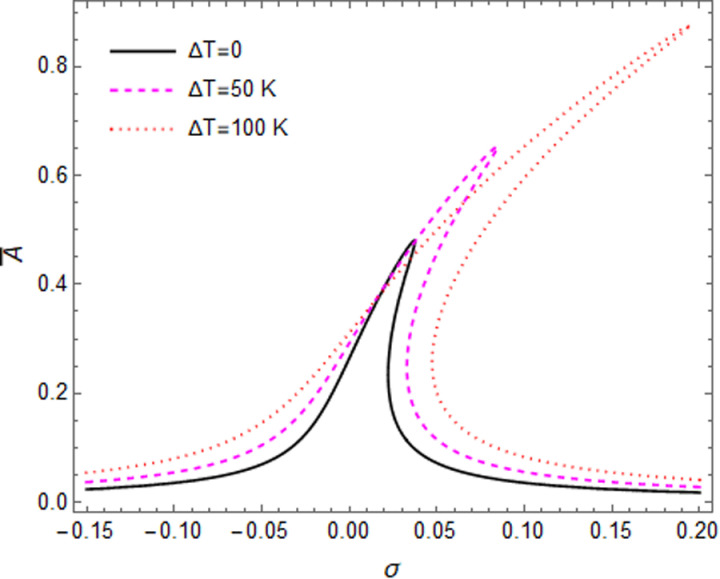
The primary resonance curves of the shell at different value of temperature rise.

Effects of temperature rise on the primary resonance response of the GPL reinforced shell are illustrated in Fig 11 when R/h = 10 and the microshell fundamental mode occurred at n = 2. It can be seen that the temperature rise, increases the hardening effects and amplitude of the peak point, since the rise of temperature leads to reduction of the shell stiffness.

## 4 Conclusions

This paper investigates the size-dependent nonlinear forced vibration behavior of FG-GPLRC shells using MCST under thermal conditions, considering three different FG-GPL distributions through the thickness. The main findings are as follows: All primary resonance curves shift to the right, indicating hardening stiffness behavior. The small-scale parameter amplifies the nonlinearity. Increasing the volume fraction of GPLs in the matrix, especially near the inner and outer surfaces of the shell, enhances the stiffness and reduces the nonlinearity of the reinforced composite. As the temperature rises, the amplitude of vibration increases, along with the nonlinear hardening characteristics of the shell. Moreover, increasing the h/Rh/Rh/R ratio of the cylindrical shell, while maintaining the fundamental mode shape, further intensifies the hardening effects.

## Appendix A


$$C_{11}=\left(-\frac{A_{11}m^{2}\pi^{3}}{2L}-\frac{A_{66}Ln^{2}\pi }{2R^{2}}-\left(\frac{Ln^{2}\pi \mu_{0}l^{2}}{2R^{4}}+\frac{Ln^{4}\pi \mu_{0}l^{2}}{8R^{4}}+\frac{m^{2}n^{2}\pi^{3}\mu_{0}l^{2}}{8LR^{2}}\right)\right);$$

$$C_{12}=\left(-\frac{A_{12}mn\pi^{2}}{2R}-\frac{A_{66}mn\pi^{2}}{2R}+\left(\frac{mn\pi^{2}\mu_{0}l^{2}}{8R^{3}}+\frac{mn^{3}\pi^{2}\mu_{0}l^{2}}{8R^{3}}+\frac{m^{3}n\pi^{4}\mu_{0}l^{2}}{8L^{2}R}\right)\right);$$

$$C_{13}=\left(\frac{A_{12}m\pi^{2}}{2R}-\frac{mn^{2}\pi^{2}\mu_{0}l^{2}}{4R^{3}}\right);$$

$$C_{14}=\left(-\frac{B_{11}m^{2}\pi^{3}}{2L}-\frac{B_{66}Ln^{2}\pi }{2R^{2}}-\left(-\frac{5Ln^{2}\pi \mu_{0}l^{2}}{8R^{3}}+\frac{Ln^{4}\pi \mu_{1}l^{2}}{8R^{4}}+\frac{m^{2}n^{2}\pi^{3}\mu_{1}l^{2}}{8LR^{2}}\right)\right);$$

$$C_{15}=(-\frac{B_{12}mn\pi^{2}}{2R}-\frac{B_{66}mn\pi^{2}}{2R}-(\frac{mn\pi^{2}\mu_{0}l^{2}}{8R^{2}}-\frac{mn\pi^{2}\mu_{1}l^{2}}{4R^{3}}-\frac{mn^{3}\pi^{2}\mu_{1}l^{2}}{8R^{3}}-\frac{m^{3}n\pi^{4}\mu_{1}l^{2}}{8L^{2}R}));$$

$$C_{21}=\left(-\frac{A_{12}mn\pi^{2}}{2R}-\frac{A_{66}mn\pi^{2}}{2R}+\left(\frac{mn\pi^{2}\mu_{0}l^{2}}{8R^{3}}+\frac{mn^{3}\pi^{2}\mu_{0}l^{2}}{8R^{3}}+\frac{m^{3}n\pi^{4}\mu_{0}l^{2}}{8L^{2}R}\right)\right);$$

$$C_{22}=\left(-\frac{A_{66}m^{2}\pi^{3}}{2L}-\frac{A_{22}Ln^{2}\pi }{2R^{2}}-\left(\frac{m^{4}\pi^{5}\mu_{0}l^{2}}{8L^{3}}+\frac{Ln^{2}\pi \mu_{0}l^{2}}{8R^{4}}+\frac{m^{2}\pi^{3}\mu_{0}l^{2}}{4LR^{2}}+\frac{m^{2}n^{2}\pi^{3}\mu_{0}l^{2}}{8LR^{2}}\right)\right);$$

$$C_{23}=\left(\frac{A_{22}Ln\pi }{2R^{2}}+\frac{E_{55}Ln\pi }{2R^{2}}+\left(\frac{Ln\pi \mu_{0}l^{2}}{8R^{4}}+\frac{Ln^{3}\pi \mu_{0}l^{2}}{8R^{4}}+\frac{3m^{2}n\pi^{3}\mu_{0}l^{2}}{8LR^{2}}\right)\right);$$

$$C_{24}=\left(-\frac{B_{12}mn\pi^{2}}{2R}-\frac{B_{66}mn\pi^{2}}{2R}-\left(\frac{mn\pi^{2}\mu_{0}l^{2}}{4R^{2}}+\frac{mn\pi^{2}\mu_{1}l^{2}}{8R^{3}}-\frac{mn^{3}\pi^{2}\mu_{1}l^{2}}{8R^{3}}-\frac{m^{3}n\pi^{4}\mu_{1}l^{2}}{8L^{2}R}\right)\right);$$

$$C_{25}=(-\frac{B_{66}m^{2}\pi^{3}}{2L}-\frac{B_{22}Ln^{2}\pi }{2R^{2}}-(\frac{Ln^{2}\pi \mu_{0}l^{2}}{8R^{3}}+\frac{3m^{2}\pi^{3}\mu_{0}l^{2}}{8LR}+\frac{m^{4}\pi^{5}\mu_{1}l^{2}}{8L^{3}}+\frac{m^{2}\pi^{3}\mu_{1}l^{2}}{8LR^{2}}+\frac{m^{2}n^{2}\pi^{3}\mu_{1}l^{2}}{8LR^{2}}));$$

$$C_{31}=\left(\frac{A_{21}m\pi^{2}}{2R}-\frac{mn^{2}\pi^{2}\mu_{0}l^{2}}{4R^{3}}\right);$$

$$C_{32}=\left(\frac{A_{22}Ln\pi }{2R^{2}}+\frac{E_{55}Ln\pi }{2R^{2}}+\left(\frac{Ln\pi \mu_{0}l^{2}}{8R^{4}}+\frac{Ln^{3}\pi \mu_{0}l^{2}}{8R^{4}}+\frac{3m^{2}n\pi^{3}\mu_{0}l^{2}}{8LR^{2}}\right)\right);$$

$$C_{33}=(-\frac{E_{44}m^{2}\pi^{3}}{2L}+\frac{m^{2}N_{1}^{T}\pi^{3}}{2L}-\frac{A_{22}L\pi }{2R^{2}}-\frac{E_{55}Ln^{2}\pi }{2R^{2}}+\frac{Ln^{2}N_{2}^{T}\pi }{2R^{2}}-\left(\frac{m^{4}\pi^{5}\mu_{0}l^{2}}{8L^{3}}+\frac{Ln^{2}\pi \mu_{0}l^{2}}{8R^{4}}+\frac{Ln^{4}\pi \mu_{0}l^{2}}{8R^{4}}+\frac{m^{2}\pi^{3}\mu_{0}l^{2}}{8LR^{2}}+\frac{m^{2}n^{2}\pi^{3}\mu_{0}l^{2}}{4LR^{2}}\right));$$

$$C_{34}=\left(-\frac{1}{2}E_{55}m\pi^{2}+\frac{B_{21}m\pi^{2}}{2R}+\left(\frac{m^{3}\pi^{4}\mu_{0}l^{2}}{8L^{2}}+\frac{m\pi^{2}\mu_{0}l^{2}}{8R^{2}}+\frac{mn^{2}\pi^{2}\mu_{0}l^{2}}{8R^{2}}\right)\right);$$

$$C_{35}=\left(\frac{B_{22}Ln\pi }{2R^{2}}-\frac{E_{55}Ln\pi }{2R}+\left(-\frac{Ln\pi \mu_{0}l^{2}}{8R^{3}}+\frac{Ln^{3}\pi \mu_{0}l^{2}}{8R^{3}}+\frac{m^{2}n\pi^{3}\mu_{0}l^{2}}{8LR}+\frac{m^{2}n\pi^{3}\mu_{1}l^{2}}{4LR^{2}}\right)\right);$$

$$C_{36}=\frac{1}{2}\left(-\frac{27A_{11}m^{4}\pi^{5}}{32L^{3}}-\frac{27A_{22}Ln^{4}\pi }{32R^{4}}-\frac{3A_{12}m^{2}n^{2}\pi^{3}}{32LR^{2}}-\frac{3A_{21}m^{2}n^{2}\pi^{3}}{32LR^{2}}-\frac{3A_{66}m^{2}n^{2}\pi^{3}}{8LR^{2}}\right);$$

$$C_{37}=\frac{1}{6}(-\frac{27A_{11}m^{4}\pi^{5}}{32L^{3}}-\frac{27A_{22}Ln^{4}\pi }{32R^{4}}-\frac{3A_{12}m^{2}n^{2}\pi^{3}}{32LR^{2}}-\frac{3A_{21}m^{2}n^{2}\pi^{3}}{32LR^{2}}-\frac{3A_{66}m^{2}n^{2}\pi^{3}}{8LR^{2}});$$

$$C_{41}=\left(-\frac{B_{11}m^{2}\pi^{3}}{2L}-\frac{B_{66}Ln^{2}\pi }{2R^{2}}-\left(-\frac{5Ln^{2}\pi \mu_{0}l^{2}}{8R^{3}}+\frac{Ln^{4}\pi \mu_{1}l^{2}}{8R^{4}}+\frac{m^{2}n^{2}\pi^{3}\mu_{1}l^{2}}{8LR^{2}}\right)\right);$$

$$C_{42}=\left(-\frac{B_{12}mn\pi^{2}}{2R}-\frac{B_{66}mn\pi^{2}}{2R}-\left(\frac{mn\pi^{2}\mu_{0}l^{2}}{4R^{2}}+\frac{mn\pi^{2}\mu_{1}l^{2}}{8R^{3}}-\frac{mn^{3}\pi^{2}\mu_{1}l^{2}}{8R^{3}}-\frac{m^{3}n\pi^{4}\mu_{1}l^{2}}{8L^{2}R}\right)\right);$$

$$C_{43}=\left(-\frac{1}{2}E_{44}m\pi^{2}+\frac{B_{12}m\pi^{2}}{2R}+\left(\frac{m^{3}\pi^{4}\mu_{0}l^{2}}{8L^{2}}+\frac{m\pi^{2}\mu_{0}l^{2}}{8R^{2}}+\frac{mn^{2}\pi^{2}\mu_{0}l^{2}}{8R^{2}}\right)\right);$$

$$C_{44}=\left(-\frac{D_{11}m^{2}\pi^{3}}{2L}-\frac{D_{66}Ln^{2}\pi }{2R^{2}}-\left(\frac{m^{2}\pi^{3}\mu_{0}l^{2}}{8L}+\frac{Ln^{2}\pi \mu_{0}l^{2}}{2R^{2}}-\frac{Ln^{2}\pi \mu_{1}l^{2}}{4R^{3}}+\frac{Ln^{4}\pi \mu_{2}l^{2}}{8R^{2}}+\frac{m^{2}n^{2}\pi^{3}\mu_{2}l^{2}}{8LR^{2}}\right)\right);$$

$$C_{45}=(-\frac{D_{12}mn\pi^{2}}{2R}-\frac{D_{66}mn\pi^{2}}{2R}+(\frac{3mn\pi^{2}\mu_{0}l^{2}}{8R}-\frac{mn\pi^{2}\mu_{1}l^{2}}{4R^{2}}+\frac{mn^{3}\pi^{2}\mu_{2}l^{2}}{8R^{3}}+\frac{m^{3}n\pi^{4}\mu_{2}l^{2}}{8L^{2}R}));$$

$$C_{51}=\left(-\frac{B_{21}mn\pi^{2}}{2R}-\frac{B_{66}mn\pi^{2}}{2R}+\left(-\frac{mn\pi^{2}\mu_{0}l^{2}}{8R^{2}}+\frac{mn\pi^{2}\mu_{1}l^{2}}{4R^{3}}+\frac{mn^{3}\pi^{2}\mu_{1}l^{2}}{8R^{3}}+\frac{m^{3}n\pi^{4}\mu_{1}l^{2}}{8L^{2}R}\right)\right);$$

$$C_{52}=\left(-\frac{B_{66}m^{2}\pi^{3}}{2L}-\frac{B_{22}Ln^{2}\pi }{2R^{2}}-\left(\frac{Ln^{2}\pi \mu_{0}l^{2}}{8R^{3}}+\frac{3m^{2}\pi^{3}\mu_{0}l^{2}}{8LR}+\frac{m^{4}\pi^{5}\mu_{1}l^{2}}{8L^{3}}+\frac{m^{2}\pi^{3}\mu_{1}l^{2}}{8LR^{2}}+\frac{m^{2}n^{2}\pi^{3}\mu_{1}l^{2}}{8LR^{2}}\right)\right);$$

$$C_{53}=\left(\frac{B_{22}Ln\pi }{2R^{2}}-\frac{E_{55}Ln\pi }{2R}+\left(-\frac{Ln\pi \mu_{0}l^{2}}{8R^{3}}+\frac{Ln^{3}\pi \mu_{0}l^{2}}{8R^{3}}+\frac{m^{2}n\pi^{3}\mu_{0}l^{2}}{8LR}+\frac{m^{2}n\pi^{3}\mu_{1}l^{2}}{4LR^{2}}\right)\right);$$

$$C_{54}=\left(-\frac{D_{12}mn\pi^{2}}{2R}-\frac{D_{66}mn\pi^{2}}{2R}+\left(\frac{3mn\pi^{2}\mu_{0}l^{2}}{8R}-\frac{mn\pi^{2}\mu_{1}l^{2}}{4R^{2}}+\frac{mn^{3}\pi^{2}\mu_{2}l^{2}}{8R^{3}}+\frac{m^{3}n\pi^{4}\mu_{2}l^{2}}{8L^{2}R}\right)\right);$$

$$C_{55}=(-\frac{D_{66}m^{2}\pi^{3}}{2L}-\frac{D_{22}Ln^{2}\pi }{2R^{2}}-(\frac{m^{2}\pi^{3}\mu_{0}l^{2}}{2L}+\frac{Ln^{2}\pi \mu_{0}l^{2}}{8R^{2}}+\frac{m^{2}\pi^{3}\mu_{1}l^{2}}{4LR}+\frac{m^{4}\pi^{5}\mu_{2}l^{2}}{8L^{3}}+\frac{m^{2}\pi^{3}\mu_{2}l^{2}}{4LR^{2}}+\frac{m^{2}n^{2}\pi^{3}\mu_{2}l^{2}}{8LR^{2}}));$$

### Appendix B

*K*_*iw*_ and *K*_*iww*_; (i = u, v, x, y) in Eq. (33) can be written as:


$$K_{uw}=\left[\begin{array}{c} C_{53}\left(-C_{12}C_{24}C_{45}+C_{12}C_{25}C_{44}+C_{14}C_{22}C_{45}-C_{14}C_{25}C_{42}-C_{15}C_{22}C_{44}+C_{15}C_{24}C_{42}\right)\\ +C_{43}\left(C_{12}C_{24}C_{55}-C_{12}C_{25}C_{54}-C_{14}C_{22}C_{55}+C_{14}C_{25}C_{52}+C_{15}C_{22}C_{54}-C_{15}C_{24}C_{52}\right)\\ {+C}_{23}\left(-C_{12}C_{44}C_{55}+C_{12}C_{45}C_{54}+C_{14}C_{42}C_{55}-C_{14}C_{45}C_{52}-C_{15}C_{42}C_{54}+C_{15}C_{44}C_{52}\right)\\ {+C}_{13}\left(C_{22}C_{44}C_{55}-C_{22}C_{45}C_{54}-C_{24}C_{42}C_{55}+C_{24}C_{45}C_{52}+C_{25}C_{42}C_{54}-C_{25}C_{44}C_{52}\right) \end{array}\right]/\Delta C$$

$$K_{uww}=\left[\begin{array}{c} C_{56}\left(-C_{12}C_{24}C_{45}+C_{12}C_{25}C_{44}+C_{14}C_{22}C_{45}-C_{14}C_{25}C_{42}-C_{15}C_{22}C_{44}+C_{15}C_{24}C_{42}\right)\\ +C_{46}\left(C_{12}C_{24}C_{55}-C_{12}C_{25}C_{54}-C_{14}C_{22}C_{55}+C_{14}C_{25}C_{52}+C_{15}C_{22}C_{54}-C_{15}C_{24}C_{52}\right)\\ {+C}_{26}\left(-C_{12}C_{44}C_{55}+C_{12}C_{45}C_{54}+C_{14}C_{42}C_{55}-C_{14}C_{45}C_{52}-C_{15}C_{42}C_{54}+C_{15}C_{44}C_{52}\right)\\ {+C}_{16}\left(C_{22}C_{44}C_{55}-C_{22}C_{45}C_{54}-C_{24}C_{42}C_{55}+C_{24}C_{45}C_{52}+C_{25}C_{42}C_{54}-C_{25}C_{44}C_{52}\right) \end{array}\right]/\Delta C$$

$$K_{vw}=\left[\begin{array}{c} C_{53}\left(C_{11}C_{24}C_{45}-C_{11}C_{25}C_{44}-C_{14}C_{21}C_{45}+C_{14}C_{25}C_{41}+C_{15}C_{21}C_{44}+C_{15}C_{24}C_{41}\right)\\ +C_{43}\left({-C}_{11}C_{24}C_{55}+C_{11}C_{25}C_{54}+C_{14}C_{21}C_{55}-C_{14}C_{25}C_{51}-C_{15}C_{21}C_{54}+C_{15}C_{24}C_{51}\right)\\ {+C}_{23}\left(C_{11}C_{44}C_{55}-C_{11}C_{45}C_{54}-C_{14}C_{41}C_{55}+C_{14}C_{45}C_{51}+C_{15}C_{41}C_{54}-C_{15}C_{44}C_{51}\right)\\ {+C}_{13}\left(-C_{21}C_{44}C_{55}+C_{21}C_{45}C_{54}+C_{24}C_{41}C_{55}-C_{24}C_{45}C_{51}-C_{25}C_{41}C_{54}+C_{25}C_{44}C_{51}\right) \end{array}\right]/\Delta C$$

$$K_{vww}=\left[\begin{array}{c} C_{56}\left(C_{11}C_{24}C_{45}-C_{11}C_{25}C_{44}-C_{14}C_{21}C_{45}+C_{14}C_{25}C_{41}+C_{15}C_{21}C_{44}-C_{15}C_{24}C_{41}\right)\\ +C_{46}\left({-C}_{11}C_{24}C_{55}+C_{11}C_{25}C_{54}+C_{14}C_{21}C_{55}-C_{14}C_{25}C_{51}-C_{15}C_{21}C_{54}+C_{15}C_{24}C_{51}\right)\\ {+C}_{26}\left(C_{11}C_{44}C_{55}-C_{11}C_{45}C_{54}-C_{14}C_{41}C_{55}+C_{14}C_{45}C_{51}+C_{15}C_{41}C_{54}-C_{15}C_{44}C_{51}\right)\\ {+C}_{16}\left(-C_{21}C_{44}C_{55}+C_{21}C_{45}C_{54}+C_{24}C_{41}C_{55}-C_{24}C_{45}C_{51}-C_{25}C_{41}C_{54}+C_{25}C_{44}C_{51}\right) \end{array}\right]/\Delta C$$

$$K_{xw}=\left[\begin{array}{c} C_{53}\left(-C_{11}C_{22}C_{45}+C_{11}C_{25}C_{42}+C_{12}C_{21}C_{45}-C_{12}C_{25}C_{41}-C_{15}C_{21}C_{42}+C_{15}C_{22}C_{41}\right)\\ +C_{43}\left(C_{11}C_{22}C_{55}-C_{11}C_{25}C_{52}-C_{12}C_{21}C_{55}+C_{12}C_{25}C_{51}+C_{15}C_{21}C_{52}-C_{15}C_{22}C_{51}\right)\\ {+C}_{23}\left({-C}_{11}C_{42}C_{55}+C_{11}C_{45}C_{52}+C_{12}C_{41}C_{55}-C_{12}C_{45}C_{51}-C_{15}C_{41}C_{52}+C_{15}C_{42}C_{51}\right)\\ {+C}_{13}\left(C_{21}C_{42}C_{55}-C_{21}C_{45}C_{52}-C_{22}C_{41}C_{55}+C_{22}C_{45}C_{51}+C_{25}C_{41}C_{54}-C_{25}C_{42}C_{51}\right) \end{array}\right]/\Delta C$$

$$K_{xww}=\left[\begin{array}{c} C_{56}\left(-C_{11}C_{22}C_{45}+C_{11}C_{25}C_{42}+C_{12}C_{21}C_{45}-C_{12}C_{25}C_{41}-C_{15}C_{21}C_{42}+C_{15}C_{22}C_{41}\right)\\ +C_{46}\left(C_{11}C_{22}C_{55}-C_{11}C_{25}C_{52}-C_{12}C_{21}C_{55}+C_{12}C_{25}C_{51}+C_{15}C_{21}C_{52}-C_{15}C_{22}C_{51}\right)\\ {+C}_{26}\left({-C}_{11}C_{42}C_{55}+C_{11}C_{45}C_{52}+C_{12}C_{41}C_{55}-C_{12}C_{45}C_{51}-C_{15}C_{41}C_{52}+C_{15}C_{42}C_{51}\right)\\ {+C}_{16}\left(C_{21}C_{42}C_{55}-C_{21}C_{45}C_{52}-C_{22}C_{41}C_{55}+C_{22}C_{45}C_{51}+C_{25}C_{41}C_{54}-C_{25}C_{42}C_{51}\right) \end{array}\right]/\Delta C$$

$$K_{yw}=\left[\begin{array}{c} C_{53}\left(C_{11}C_{22}C_{44}-C_{11}C_{24}C_{42}-C_{12}C_{21}C_{44}+C_{12}C_{24}C_{41}+C_{14}C_{21}C_{42}-C_{14}C_{22}C_{41}\right)\\ +C_{43}\left({-C}_{11}C_{22}C_{54}+C_{11}C_{24}C_{52}+C_{12}C_{21}C_{54}-C_{12}C_{24}C_{51}+C_{14}C_{21}C_{52}+C_{14}C_{22}C_{51}\right)\\ {+C}_{23}\left(C_{11}C_{42}C_{54}-C_{11}C_{44}C_{52}-C_{12}C_{41}C_{54}+C_{12}C_{44}C_{51}+C_{14}C_{41}C_{52}-C_{14}C_{42}C_{51}\right)\\ {+C}_{13}\left(-C_{21}C_{42}C_{54}+C_{21}C_{44}C_{52}+C_{22}C_{41}C_{54}-C_{22}C_{44}C_{51}-C_{24}C_{41}C_{52}+C_{24}C_{42}C_{51}\right) \end{array}\right]/\Delta C$$

$$K_{yww}=\left[\begin{array}{c} C_{56}\left(C_{11}C_{22}C_{44}-C_{11}C_{24}C_{42}-C_{12}C_{21}C_{44}+C_{12}C_{24}C_{41}+C_{14}C_{21}C_{42}-C_{14}C_{22}C_{41}\right)\\ +C_{46}\left({-C}_{11}C_{22}C_{54}+C_{11}C_{24}C_{52}+C_{12}C_{21}C_{54}-C_{12}C_{24}C_{51}+C_{14}C_{21}C_{52}+C_{14}C_{22}C_{51}\right)\\ {+C}_{26}\left(\left(C_{11}C_{42}C_{54}-C_{11}C_{44}C_{52}-C_{12}C_{41}C_{54}+C_{12}C_{44}C_{51}+C_{14}C_{41}C_{52}-C_{14}C_{42}C_{51}\right)\right)\\ {+C}_{16}\left(-C_{21}C_{42}C_{54}+C_{21}C_{44}C_{52}+C_{22}C_{41}C_{54}-C_{22}C_{44}C_{51}-C_{24}C_{41}C_{52}+C_{24}C_{42}C_{51}\right) \end{array}\right]/\Delta C$$

$$\Delta C=\left[\begin{array}{c} C_{11}\left(C_{55}C_{22}C_{44}-C_{22}C_{45}C_{54}-C_{24}C_{42}C_{55}+C_{24}C_{45}C_{52}+C_{25}C_{42}C_{54}-C_{25}C_{44}C_{52}\right)\\ +C_{12}\left({-C}_{55}C_{21}C_{44}+C_{21}C_{45}C_{54}+C_{24}C_{41}C_{55}-C_{24}C_{45}C_{51}-C_{25}C_{41}C_{54}+C_{25}C_{44}C_{51}\right)\\ +C_{14}\left(C_{55}C_{21}C_{42}-C_{21}C_{45}C_{52}-C_{22}C_{41}C_{55}+C_{22}C_{45}C_{51}+C_{25}C_{41}C_{52}-C_{25}C_{42}C_{51}\right)\\ +C_{15}\left(-C_{54}C_{21}C_{42}+C_{21}C_{44}C_{52}+C_{22}C_{41}C_{54}-C_{22}C_{44}C_{51}-C_{24}C_{41}C_{52}+C_{24}C_{42}C_{51}\right) \end{array}\right]$$

## References

[1] Sun J, et al. 0.79 ppm scale-factor nonlinearity whole-angle microshell gyroscope realized by real-time calibration of capacitive displacement detection. Microsyst Nanoeng. 2021;7(1):79. 34721887 10.1038/s41378-021-00306-6 PMC8514555

[2] Shi Y, Lu K, Xi X, Wu Y, Xiao D, Wu X. Geometric imperfection characterization and precise assembly of micro shell resonators. J Microelectromech Syst. 2020;29(4):480–9.

[3] Huang X, Qi X, Boey F, Zhang H. Graphene-based composites. Chem Soc Rev. 2012;41(2):666–86. doi: 10.1039/c1cs15078b 21796314

[4] Kim H, Abdala AA, Macosko CW. Graphene/Polymer Nanocomposites. Macromolecules. 2010;43(16):6515–30. doi: 10.1021/ma100572e

[5] Rafiee M, Rafiee J, Yu Z, Koratkar N. Buckling resistant graphene nanocomposites. Appl Phys Lett. 2009;95(22):223103.

[6] Rafiee MA, Rafiee J, Wang Z, Song H, Yu Z-Z, Koratkar N. Enhanced mechanical properties of nanocomposites at low graphene content. ACS Nano. 2009;3(12):3884–90. doi: 10.1021/nn9010472 19957928

[7] Li F-M, Yao G. 1/3 Subharmonic resonance of a nonlinear composite laminated cylindrical shell in subsonic air flow. Composite Structures. 2013;100:249–56. doi: 10.1016/j.compstruct.2012.12.035

[8] Jafari AA, Khalili SMR, Tavakolian M. Nonlinear vibration of functionally graded cylindrical shells embedded with a piezoelectric layer. Thin-Walled Structures. 2014;79:8–15. doi: 10.1016/j.tws.2014.01.030

[9] Hosseini-Hashemi Sh, Abaei AR, Ilkhani MR. Free vibrations of functionally graded viscoelastic cylindrical panel under various boundary conditions. Composite Structures. 2015;126:1–15. doi: 10.1016/j.compstruct.2015.02.031

[10] Soltanimaleki A, Foroutan M, Alihemmati J. Free vibration analysis of functionally graded fiber reinforced cylindrical panels by a three dimensional mesh-free model. Journal of Vibration and Control. 2016;22(19):4087–98. doi: 10.1177/1077546315570717

[11] Shen H-S, Xiang Y, Fan Y. Nonlinear vibration of functionally graded graphene-reinforced composite laminated cylindrical shells in thermal environments. Compos Struct. 2017;182:447–56.

[12] Dong YH, Li YH, Chen D, Yang J. Vibration characteristics of functionally graded graphene reinforced porous nanocomposite cylindrical shells with spinning motion. Composites Part B: Engineering. 2018;145:1–13. doi: 10.1016/j.compositesb.2018.03.009

[13] Niu Y, Zhang W, Guo XY. Free vibration of rotating pretwisted functionally graded composite cylindrical panel reinforced with graphene platelets. European Journal of Mechanics - A/Solids. 2019;77:103798. doi: 10.1016/j.euromechsol.2019.103798

[14] Arani AG, Kiani F, Afshari H. Free and forced vibration analysis of laminated functionally graded CNT-reinforced composite cylindrical panels. Jnl of Sandwich Structures & Materials. 2019;23(1):255–78. doi: 10.1177/1099636219830787

[15] Barati MR, Zenkour AM. Vibration analysis of functionally graded graphene platelet reinforced cylindrical shells with different porosity distributions. Mechanics of Advanced Materials and Structures. 2018;26(18):1580–8. doi: 10.1080/15376494.2018.1444235

[16] Bahaadini R, Saidi AR, Arabjamaloei Z, Ghanbari-Nejad-Parizi A. Vibration Analysis of Functionally Graded Graphene Reinforced Porous Nanocomposite Shells. Int J Appl Mechanics. 2019;11(07):1950068. doi: 10.1142/s1758825119500686

[17] Wang YQ, Ye C, Zu JW. Nonlinear vibration of metal foam cylindrical shells reinforced with graphene platelets. Aerospace Science and Technology. 2019;85:359–70. doi: 10.1016/j.ast.2018.12.022

[18] Wu Z, Zhang Y, Yao G. Nonlinear forced vibration of functionally graded carbon nanotube reinforced composite circular cylindrical shells. Acta Mech. 2020;231(6):2497–519. doi: 10.1007/s00707-020-02650-6

[19] Yadav A, Amabili M, Panda SK, Dey T, Kumar R. Forced nonlinear vibrations of circular cylindrical sandwich shells with cellular core using higher-order shear and thickness deformation theory. Journal of Sound and Vibration. 2021;510:116283. doi: 10.1016/j.jsv.2021.116283

[20] Teng MW, Wang YQ. Spin-induced internal resonance in circular cylindrical shells. International Journal of Non-Linear Mechanics. 2022;147:104234. doi: 10.1016/j.ijnonlinmec.2022.104234

[21] Dong Y, Hu H, Wang L. A comprehensive study on the coupled multi-mode vibrations of cylindrical shells. Mech Syst Signal Process. 2022;169(November 2021):1–25.

[22] Wang G, Zhu Z, Zhang Y, Xu R, Jiang Y, Liu Q. Free and forced vibration analysis of thin-walled cylindrical shells with arbitrary boundaries in steady thermal environment. Thin-Walled Structures. 2023;185:110556. doi: 10.1016/j.tws.2023.110556

[23] Zhang Y-W, She G-L. Nonlinear primary resonance of axially moving functionally graded cylindrical shells in thermal environment. Mechanics of Advanced Materials and Structures. 2023;31(16):3617–29. doi: 10.1080/15376494.2023.2180556

[24] Ali A, Hasan H. Nonlinear dynamic stability of an imperfect shear deformable orthotropic functionally graded material toroidal shell segments under the longitudinal constant velocity. Proc Inst Mech Eng Part C J Mech Eng Sci. 2019;233(19–20):6827–50.

[25] Ali A, Hasan H. Non-linear large amplitude vibration of orthotropic FGM convex and concave toroidal shell segments including the damping effect using the shear deformation theory. Thin-Walled Struct. 2022;173(December 2021):109035.

[26] Ali A, Hasan H, Mohammed F. Nonlinear dynamic buckling of a simply supported imperfect nanocomposite shear deformable plate under the effect of in-plane velocities. Commun Nonlinear Sci Numer Simul. 2024;145.

[27] Hasan H, Ali A. Nonlinear forced vibration of functionally graded graphene-reinforced composite (FG-GRC) laminated cylindrical shells under different boundary conditions with thermal repercussions. Int J Struct Stab Dyn. 2024;24(18):2450207.

[28] Tadi Beni Y, Mehralian F, Razavi H. Free vibration analysis of size-dependent shear deformable functionally graded cylindrical shell on the basis of modified couple stress theory. Composite Structures. 2015;120:65–78. doi: 10.1016/j.compstruct.2014.09.065

[29] Gholami R, Darvizeh A, Ansari R, Sadeghi F. Vibration and buckling of first-order shear deformable circular cylindrical micro-/nano-shells based on Mindlin’s strain gradient elasticity theory. European Journal of Mechanics - A/Solids. 2016;58:76–88. doi: 10.1016/j.euromechsol.2016.01.014

[30] Tohidi H, Hosseini-Hashemi S, Maghsoudpour A, Etemadi S. Dynamic stability of fg-cnt-reinforced viscoelastic micro cylindrical shells resting on nonhomogeneous orthotropic viscoelastic medium subjected to harmonic temperature distribution and 2d magnetic field. Wind Struct. 2017;25(2):131–56.

[31] Veysi A, Shabani R, Rezazadeh G. Nonlinear vibrations of micro-doubly curved shallow shells based on the modified couple stress theory. Nonlinear Dyn. 2016;87(3):2051–65. doi: 10.1007/s11071-016-3175-5

[32] Razavi H, Babadi AF, Tadi Beni Y. Free vibration analysis of functionally graded piezoelectric cylindrical nanoshell based on consistent couple stress theory. Composite Structures. 2017;160:1299–309. doi: 10.1016/j.compstruct.2016.10.056

[33] Hasrati E, Ansari R, Torabi J. A novel numerical solution strategy for solving nonlinear free and forced vibration problems of cylindrical shells. Applied Mathematical Modelling. 2018;53:653–72. doi: 10.1016/j.apm.2017.08.027

[34] Anvari M, Mohammadimehr M, Amiri A. Vibration behavior of a micro cylindrical sandwich panel reinforced by graphene platelet. Journal of Vibration and Control. 2020;26(13–14):1311–43. doi: 10.1177/1077546319892730

[35] Bidzard A, Malekzadeh P, Mohebpour SR. A size-dependent nonlinear finite element free vibration analysis of multilayer FG-GPLRC toroidal micropanels in thermal environment. Composite Structures. 2022;279:114783. doi: 10.1016/j.compstruct.2021.114783

[36] Mosayyebi M, Ashenai Ghasemi F, Aghaee M, Vahdat M. Analytical investigation of the refined zigzag theory for electro-magneto vibration response of the viscoelastic FG-GPLRC sandwich microplates. Mechanics Based Design of Structures and Machines. 2022;51(10):5941–67. doi: 10.1080/15397734.2021.2024847

[37] Yin B, Fang J. Modified couple stress-based free vibration and dynamic response of rotating FG multilayer composite microplates reinforced with graphene platelets. Arch Appl Mech. 2022;93(3):1051–79. doi: 10.1007/s00419-022-02313-z

[38] Ma Z, Xing B, Liu J. Dynamic analysis of GPLs reinforced microcapsules subjected to moving micro/nanoparticles using mathematical modeling and deep-neural networks. Measurement. 2024;225:113940. doi: 10.1016/j.measurement.2023.113940

[39] Hung PT, Nguyen-Xuan H, Phung-Van P, Thai CH. Modified strain gradient analysis of the functionally graded triply periodic minimal surface microplate using isogeometric approach. Engineering with Computers. 2024;40(5):2877–904. doi: 10.1007/s00366-023-01942-4

[40] Hung PT, Phung-Van P, Thai CH. A refined isogeometric plate analysis of porous metal foam microplates using modified strain gradient theory. Composite Structures. 2022;289:115467. doi: 10.1016/j.compstruct.2022.115467

[41] Hung PT, Thai CH, Phung-Van P. Isogeometric bending and free vibration analyses of carbon nanotube-reinforced magneto-electric-elastic microplates using a four variable refined plate theory. Computers & Structures. 2023;287:107121. doi: 10.1016/j.compstruc.2023.107121

[42] Hung P, Phung-Van P, Thai C. Small scale thermal analysis of piezoelectric--piezomagnetic fg microplates using modified strain gradient theory. Int J Mech Mater Des. 2023;19(4):739–61.

[43] Hung PT, Thai CH, Phung-Van P. Isogeometric free vibration of honeycomb sandwich microplates with the graphene nanoplatelets reinforcement face sheets. Engineering Structures. 2024;305:117670. doi: 10.1016/j.engstruct.2024.117670

[44] Hung P, Thai C, Phung-Van P. Isogeometric free vibration of functionally graded porous magneto-electro-elastic plate reinforced with graphene platelets resting on an elastic foundation. Comput Math with Appl. 2024;169:68–87.

[45] Yang B, Yang J, Kitipornchai S. Thermoelastic analysis of functionally graded graphene reinforced rectangular plates based on 3D elasticity. Meccanica. 2016;52(10):2275–92. doi: 10.1007/s11012-016-0579-8

[46] Abolhassanpour H, Shahgholi M, Ghasemi FA, Mohamadi A. L.Nonlinear vibration analysis of an axially moving thin-walled conical shell. International Journal of Non-Linear Mechanics. 2021;134:103747. doi: 10.1016/j.ijnonlinmec.2021.103747

[47] Mester L, Klarmann S, Klinkel S. Homogenization assumptions for the two-scale analysis of first-order shear deformable shells. Comput. Mech. 2023:1–35.

[48] Ding H-X, She G-L. Nonlinear primary resonance behavior of graphene platelet-reinforced metal foams conical shells under axial motion. Nonlinear Dyn. 2023;111(15):13723–52. doi: 10.1007/s11071-023-08564-x

[49] Bich DH, Xuan Nguyen N. Nonlinear vibration of functionally graded circular cylindrical shells based on improved Donnell equations. Journal of Sound and Vibration. 2012;331(25):5488–501. doi: 10.1016/j.jsv.2012.07.024

[50] Rouche N, Habets P, Laloy M. Stability theory by liapunov’s direct method. Springer. 1977.

[51] Nayfeh JF, Rivieccio NJ. Nonlinear Vibration of Composite Shell Subjected to Resonant Excitations. J Aerosp Eng. 2000;13(2):59–68. doi: 10.1061/(asce)0893-1321(2000)13:2(59

